# Hemicellulose pyrolysis: mechanism and kinetics of functionalized xylopyranose†

**DOI:** 10.1039/d3cp06094b

**Published:** 2024-04-04

**Authors:** Leandro Ayarde-Henríquez, Jacopo Lupi, Stephen Dooley

**Affiliations:** a School of Physics, Trinity College Dublin Dublin 2 Ireland leandro.ayarde@tcd.ie stephen.dooley@tcd.ie; b AMBER, Advanced Materials and BioEngineering Research Centre Dublin 2 Ireland

## Abstract

This work analyzes the thermochemical kinetic influence of the most prominent functionalizations of the β-d-xylopyranose motif, specifically 4-methoxy, 5-carboxyl, and 2-*O*-acetyl, regarding the pyrolytic depolymerization mechanism. The gas-phase potential energy surface of the initial unimolecular decomposition reactions is computed with M06-2X/6-311++G(d,p), following which energies are refined using the G4 and CBS-QB3 composite methods. Rate constants are computed using the transition state theory. The energies are integrated within the atomization method to assess for the first time the standard enthalpy of formation of β-d-xylopyranose, 4-methoxy-5-carboxy-β-d-xylopyranose, and 2-*O*-acetyl-β-d-xylopyranose: −218.2, −263.1, and −300.0 kcal mol^−1^, respectively. For all isomers, the activation enthalpies of ring-opening are considerably lower, 43.8–47.5 kcal mol^−1^, than the ring-contraction and elimination processes, which show higher values ranging from 61.0–81.1 kcal mol^−1^. The functional groups exert a notable influence, lowering the barrier of discrete elementary reactions by 1.9–8.3 kcal mol^−1^, increasing thus the reaction rate constant by 0–4 orders of magnitude relative to unsubstituted species. Regardless of the functionalization, the ring-opening process appears to be the most kinetically favored, characterized by a rate constant on the order 10^1^ s^−1^, exceeding significantly the values associated with ring-contraction and elimination, which fall in the range 10^−4^–10^−10^ s^−1^. This analysis shows the decomposition kinetics are contingent on the functionalization specificities and the relative orientation of reacting centers. A relatively simple chemical reactivity and bonding analysis partially support the elaborated thermokinetic approach. These insights hold significance as they imply that many alternative decomposition routes can be quickly, yet accurately, informed in forthcoming explorations of potential energy surfaces of diverse hemicellulose motifs under pyrolysis conditions.

## Abstract 2

The pyrolytic conversion of β-d-xylopyranose species to acyclic compounds is the fastest process regardless of functional groups, significantly simplifying the exploration of the potential energy surface and contributing to bioproducts deployment.

## Graphical abstract

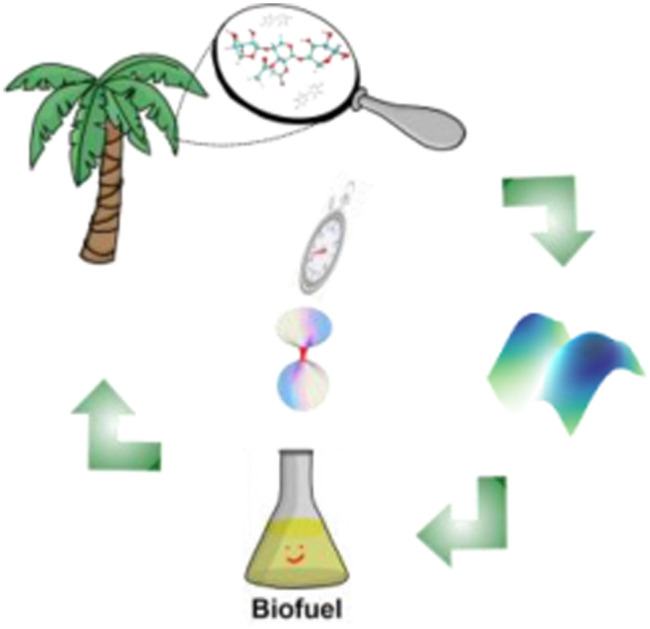

## Introduction

**Scheme 1:**
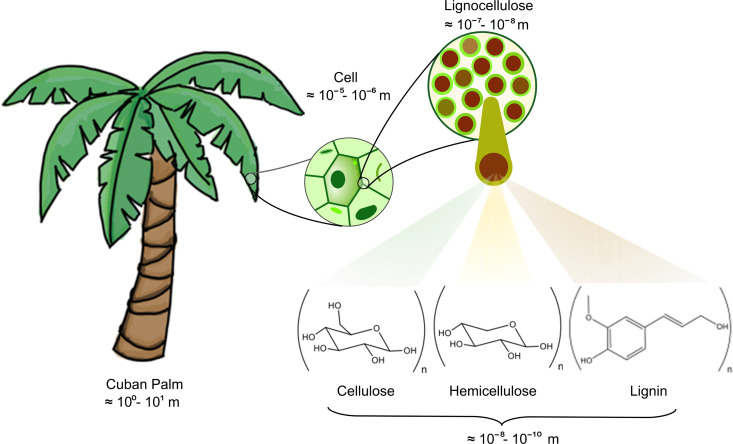
Typical structural motifs for modeling the lignocellulose (biomass) building blocks: cellulose, hemicellulose, and lignin. This graphical representation stresses the scale and multiscale challenges, which limit the current understanding of the thermal conversion of biomass into valuable bioproducts.

In contemporary global economic scenarios, predominant reliance persists on non-renewable energy resources, including coal, natural gas, and petroleum.^1,2^ Recent assessments underscore that worldwide energy consumption has reached a staggering 412 exajoules per year (EJ per year),^1^ displaying an upward trajectory for the past ten decades, barring any economic recessions. This escalating demand stresses the importance of identifying and harnessing alternative, sustainable fuel sources.^3^ Fortunately, biomass emerges as a plentiful and renewable reservoir of sustainable carbon and hydrogen with the potential to provide up to 147 EJ in 2030.^1,2^ Biomass broadly encompasses organic matter of plants and animals;^4,5^ however, this term is usually restricted to the referring of botanical constituents. Plant biomass is a composite of diverse organic compounds, including lipids, sugars, and lignocellulose, the latter being the fundamental structural material of plants. The inherent complexity of lignocellulosic plant matter is illustrated through the relative proportions of its principal components: cellulose 40–60%, hemicellulose 15–30%, and lignin 10–25%.^4,6^ Cellulose, the most abundant saccharide on Earth,^7–10^ is an ordered polymeric structure of glucose (C_6_H_12_O_6_). Frequently, cellulose is represented in terms of its basic unit, namely, the degree of polymerization (*n*), highlighting its polysaccharide nature, *i.e.*, (C_6_H_10_O_5_)_*n*_.^4,7^ This renewable and biodegradable polymer has found wide-ranging applications across diverse food, textile, and plastics industries.^7^ In contrast, hemicellulose is not a homopolysaccharide since it is formed by shorter chains, including mainly hexoses (C_6_H_12_O_6_) and pentoses (C_5_H_10_O_5_).^11,12^ Conversely, lignin presents an amorphous polymeric structure of aromatic ether rings attached to a three-carbon propene tail,^13–15^ as presented in Scheme 1.

The degradation of biomass to valuable chemicals and fuels through thermochemical processes, such as pyrolysis, gasification, hydrothermal liquefaction, and others, has captured the attention of the Industry, Government, and Academia.^3,4,6,16^ Among all methods for degrading biomass, the so-called fast pyrolysis (FP) is considered the most attractive.^1,17,18^ Under FP conditions, biomass is thermally decomposed (≈400–600 °C, ≈10^3^ °C) under an inert atmosphere, *i.e.*, without oxygen.^1,19,20^ Fast pyrolysis is regarded as a leading technology due to its technical and economic performance. In the context of the FP regime, it is essential to note that cellulose, hemicellulose, and lignin bio-oil yields exhibit characteristic values of approximately 70, 60, and 40%, respectively. However, these values are notably sensitive to variations in temperature. This means that an increase or drop in the temperature will cause the system to deviate from these thermal operation conditions, thus impacting these yields.^21,22^ Currently, the bio-oil produced falls short in quality and cost-effectiveness compared to its petroleum-derived crude oil counterparts. This discrepancy is primarily attributed to several issues that must be surmounted before the commercial-scale utilization of bio-products can be achieved:^1,4,5,17,23^ (i) the chemical structural complexity and high variability of main biomass components, (ii) challenges posed by the scale and multiscale problems, (iii) the imperative development of effective technologies for characterizing biomass products, and (iv) the pressing need for accurate and unified kinetic models to predict the type and relative abundance of bioproducts. From our perspective, it is crucial to conduct systematic theoretical studies that account for the influence of both metals and substituents due to the current experimental limitations. This rigorous approach is needed for elucidating fundamental molecular physical knowledge underpinning the thermal depolymerization of biomass, facilitating the transition towards a bio-based and circular economy.

Recently, our research group has been actively immersed in developing a detailed kinetic model for the pyrolysis of hemicellulose and lignin. This model is poised for further refinement through the Machine Learned Optimization of Chemical Kinetics (MLOCK) coded algorithm.^24^ MLOCK optimizes kinetic models to target data (*e.g.*, experimental measurements) through the generation and evaluation of sets of solutions by simultaneous perturbation of Arrhenius parameters. Machine learning and data-analysis techniques are employed to direct the search algorithm toward the ideal set of solutions that results in a kinetic model of high fidelity to the optimization targets. Furthermore, it is well known that a minor content of minerals is present within the plant tissues in the form of cations, oxides, salts, phosphates, and sulfates.^1,17^ This inorganic material encompasses a variety of minerals such as potassium (K), magnesium (Mg), sodium (Na), calcium (Ca), cobalt (Co), and copper (Cu).^1,17^ These elements significantly impact the depolymerization processes, although the content is typically low, ranging from 2–25% of the total mass.^1^ In addition, the presence of O-acetyl groups in hard and soft wood hemicellulose is well-documented.^25–27^ The precise positions of such groups, C2 and/or C3, were determined by combining nuclear magnetic resonance (NMR), thermogravimetric analysis (TGA), and chemical analysis. Separate experimental investigations have successfully isolated 4-methoxy-5-carboxy-β-d-xylopyranose (henceforth referred to as 4-*O*-methyl-d-glucuronic acid) from different hemicellulose plant sources.^28,29^

It should be emphasized that considerable research efforts have been dedicated to investigating the thermal degradation mechanism of cellulose, leading to comparatively fewer studies on the other two primary biomass components.^27^ In the existing literature, investigations addressing the depolymerization impact of substituents on the thermokinetic features of pyrolytic processes are scarce. Wang and co-workers^30^ identified some products resulting from the breakage of *O*-acetyl groups and 4-*O*-methyl-d-glucuronic acid units from xylan by combining thermogravimetric and Fourier-transform infrared spectroscopy techniques. Huang *et al.*^31^ proposed some pyrolytic reaction pathways for products of 2-*O*-acetyl-β-d-xylopyranose (hereafter termed 2-*O*-acetyl-xylose). However, to the best of our knowledge, there is a conspicuous absence of prior studies concerning the systematic assessment of the influence of functional groups on hemicellulose depolymerization, even though some studies show that the amount of *O*-acetyl groups could significantly impact the product yields and condition bio-oils acidity.^18,32–34^ Consequently, this work aims to discuss to what extent functional groups affect the pyrolytic degradation of the hemicellulose motif, β-d-xylopyranose (hereafter referred to as xylose), from both thermodynamic and kinetic viewpoints. Subsequent systematic studies will address other biomass components (*e.g.*, lignin), the organic–inorganic interaction, and the transferring of all these insights to our kinetic model.

The manuscript is organized as follows: first, the computational methods employed are outlined. Subsequent sections discuss the thermochemical and kinetics results for the initial pyrolysis steps of xylose, 2-*O*-acetyl-xylose, and 4-*O*-methyl-d-glucuronic acid. Additionally, the insights derived from the analysis of electron-density-based descriptors are discussed. The last section summarizes the main findings and delineates potential directions for future research.

## Computational methods

Geometry optimizations were performed at the M06-2X/6-311++G(d,p) level of theory using the Gaussian 16 code.^35^ This global hybrid functional is parametrized for non-metal elements and has been recommended for main-group thermochemistry and kinetic investigations.^36^ For all cases, the intrinsic reaction coordinate (IRC)^37^ was followed to ensure the transition state (TS) connects the correct reactant and product(s). As a standard procedure, the stationary points on the IRC were characterized as minima (*i.e.*, reactants and products) and first-order saddle points (TSs) based on the vibrational frequency signs. The adequacy of our single-reference-based methodology was assessed *via* the 𝒯_1_ diagnostic^38^ for key species along the pathways. The static correlation is insignificant for the investigated systems, meaning multireference methods are not required since 𝒯_1_ < 0.02. See the ESI† for further details.

Conformational analysis for critical species (*e.g.*, minima and TSs) along the IRC was conducted using the CREST program.^39^ This software combines semiempirical tight-binding methods with meta-dynamics to generate conformers thermodynamic ensembles within a relatively narrow energy window of 4.87 kcal mol^−1^. This set of structures was further optimized at the M06-2X/6-311++G(d,p) level and compared with those observed in scattering experiments to ensure the study of the correct enantiomer.

In order to obtain accurate thermochemistry values at both absolute zero and 298 K, composite model chemistry methods such as the complete basis set (CBS)^40,41^ and Gaussian-4 (G4)^42^ were employed. In these approaches, geometry optimizations and frequency calculations are performed using relatively simple and cost-effective methods, adding higher levels of theory in a stepwise manner.

Calculations of thermodynamic properties such as the Gibbs free energy (Δ*G*) and heat capacities (*C*_p_ and *C*_v_) at various temperatures were conducted in the rigid-rotor harmonic oscillator (RRHO) approximation using the Shermo 2.3.5 suite.^43^ This is a pretty flexible code that allows the explicit consideration of scaling factors of vibrational frequencies for several quantities, including the zero-point vibrational energy (ZPE), for determining thermochemical values and automatically treating the low frequencies *via* Grimme's^44^ entropy interpolation between harmonic and free-rotor approximations on an ideal gas behavior assumption. It is worth mentioning that Lu and Chen^43^ showed that applying quasi-RRHO (QRRHO) schemes for treating hindered internal rotors in relatively flexible molecules moderately impacts entropy (*S*). Based on harmonic approximation, the *C*_p_ is computed as follows:1
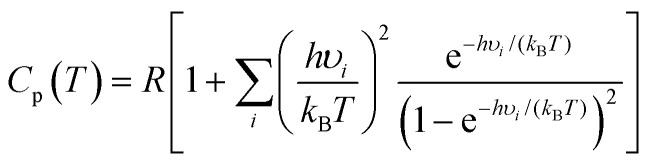
The symbols *k*_B_, *h*, and *R* are Boltzmann's constant, Planck's constant, and the universal constant of the ideal gas, respectively. The vibrational frequency of mode *i* is indicated by *υ*_*i*_, whereas *T* is the absolute temperature.

The standard enthalpy of formation, Δ*H*^0^_f_ at 298 K, of chemical species was computed using Rogers *et al.*'s methodology.^45^ This procedure combines experimental^46,47^ and computed thermochemistry data to accurately derive the values of Δ*H*^0^_f_ by using the reaction enthalpy, Δ*H*^0^_r_, as a proxy. Let us exemplify this algorithm by considering the formation of one molecule of water from its constituents (atoms) in their standard state:2
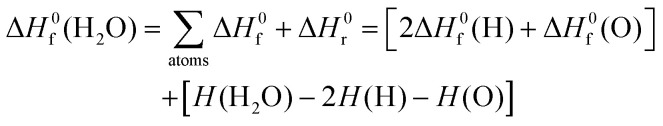
In eqn (2), the first squared bracket comprises the experiment-derived quantities reported in the Active Thermochemical Tables (ATcT).^46,47^ In contrast, the second one encapsulates the parameters to be computed using sophisticated suites such as Gaussian.^35^

The rate constant, *k*(*T*), of each elementary step is determined through canonical transition state theory (TST):3
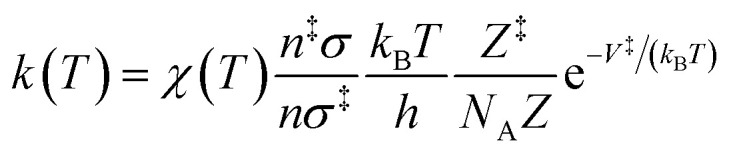
where *N*_A_ represents Avogadro's number, *n* and *n*^‡^ represent the number of chiral isomers of the reactant(s) and the transition state, respectively, while *σ* and *σ*^‡^ are the corresponding rotational symmetry numbers of these states. Moreover, the difference in the potential energy, *V*^‡^, between the transition state and reactant(s) does not include zero-point contributions as these are considered in their corresponding partition function, *Z*^‡^ and *Z*, respectively. Tunneling effect and non-classical reflection are included in the coefficient of transmission, *χ*(*T*), through Eckart's model. Furthermore, temperature-dependent rate constants were fitted to a two-parameter Arrhenius equation:4
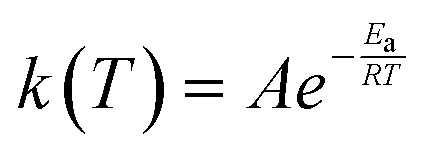
where *A* is the pre-exponent or frequency factor, and *E*_a_ is the activation energy. The kinetic analysis was conducting using the KiSThelP2021 code.^48^

The chemical reactivity assessment was conducted by calculating the electronic chemical potential, *μ*,^49^ and Fukui's functions (*f*^+^, *f*^−^, and *f*^0^).^50^ The parameter *μ* measures the global tendency of electrons to escape from a ground-state system.^49,51^ Conversely, the *f* indices describe the local response of the molecule to nucleophilic (*f*^+^), electrophilic (*f*^−^), and neutral/radical (*f*^0^) attacks.^50,52–54^ Additionally, the bonding analysis was performed using the electron localization function (ELF).^55,56^ ELF is frequently interpreted as a local measure of electrons’ kinetic energy excess due to Pauli's exclusion principle;^57,58^ thus, high values of this function (>0.5) correspond to a spatial position with high electron localization.^59,60^ The nullity condition applied to the ELF gradient yields a partition of the molecular space into basins of attractors, empirically associated with Lewis’ bonding elements, including valence bonds, lone pairs, and atomic cores.^61–63^ The numerical computation of the chemical reactivity and bonding analysis was carried out using the Multiwfn packages.^64^

## Results and discussion

### Thermochemical insights

**Scheme 2:**
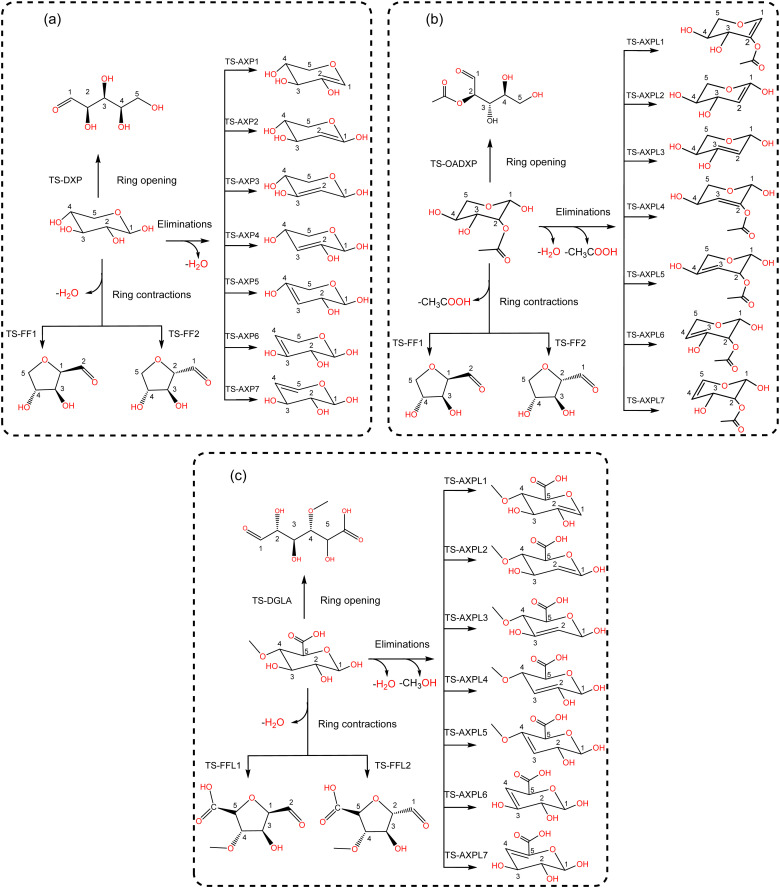
Lewis-like structures illustrating a simple kinetic model comprising key initial depolymerization reactions^65^ of xylose (panel a). Functionalized chemical species 2-*O*-acetyl-xylose (panel b) and 4-*O*-methyl-d-glucuronic acid (panel c) undergo the same reactions to systematically assess the influence of substituents. For all systems, the ring-opening process comprises the C1–O bond cleavage leading to an acyclic form, the ring-contraction involves the transformation of the pyran ring to a five-membered ring, and anhydroxylopyranose forms through elimination.

Various structural motifs have been used to investigate the depolymerization of hemicellulose under different experimental setups.^5^ Lu *et al.*^65^ extensively studied the pyrolytic mechanism of xylose, 1,4-β-d-xylobiose, and xylan by combining experimental and computational methodologies. They employed a gas-chromatograph (GC) coupled to a mass spectrometer (MS) to analyze and identify the products generated upon pyrolysis of these hemicellulose motifs. Next, a detailed reaction network comprising about 50 processes was proposed to elucidate the depolymerization mechanism of xylose, including three elemental initial steps: ring-opening, ring-contraction, and elimination. Consequently, this system is chosen as a prototypical structure for modeling hemicellulose in this study. Our goal is to assess the impact of substituents through the use of functionalized species such as 2-*O*-acetyl-xylose and 4-*O*-methyl-d-glucuronic acid undergoing the same initial reactions, as illustrated in Scheme 2. This strategic approach provides a systematic basis for hierarchically ordering subsequent steps along the decomposition channels. The ring-opening leads to an acyclic product *via* the bond scission between the pyran-ring oxygen (O) and the carbon at position 1 (C1). Conversely, furfural (FF) and furfural-like (FFL) compounds result from the ring-contraction process through both carbon–carbon (CC) and carbon–oxygen (CO) breakages. At last, reacting species undergo eliminations at positions 1, 2, 3, and 4 upon the bond cleavage of carbons at these sites and the oxygen atom of hydroxyl, methoxy, and *O*-acetyl groups. This process leads to the formation of anhydroxylopyranose (AXP), anhydroxylopyranose-like (AXPL), acetic acid (AA), and methanol. The simplest system yields AXP, whereas the other three form upon the initial pyrolytic reactions of substituted reactants. The ring-contraction and elimination entail the transfer of the proton situated at neighboring positions to the cleaving CO bond, which emphasizes the intricacies of such elementary steps.

Table 1 shows the relative enthalpy of activation, Δ*H*^‡^, at both 0 and 298 K of substituted and unsubstituted species concerning the ring opening/contraction and elimination processes at three levels of theory: M06-2X/6-311++G(d,p), CBS-QB3, and G4. The performance of G4, the highest level, in predicting activation barriers has been extensively documented for reacting systems in the gas phase. Curtiss and co-workers^66^ reported a mean unsigned deviation of 1.36 kcal mol^−1^ between predicted and experimentally derived barriers (both forward and reverse) across thirty-eight hydrogen and non-hydrogen transfer reactions. Sun and McKee^67^ investigated the initial steps of diborane pyrolysis, noting a deviation of 0.35 kcal mol^−1^ in the activation energy from experimental values regarding both uni- and bi-molecular paths. Additionally, Houk and collaborators^68^ computed barriers for 1,3-dipolar cycloadditions of ozone with low-molecular-weight hydrocarbons, demonstrating good agreement with the referential level CCSD(T)/aug-cc-pV*X*Z//CCSD(T)/cc-pVTZ, where *X* = T, Q, 5. Using reliable data, Karton and Goerigk^69^ benchmarked twenty-six pericyclic reactions, including electrocyclic, sigmatropic shift, cycloreversion, and cycloaddition subclasses, reporting a mean absolute deviation equal to 0.6 kcal mol^−1^. These studies evidence the suitability of G4 in generating accurate thermodynamic properties, particularly activation enthalpies.

**Table 1:** Activation enthalpies, Δ*H*^‡^, in kcal mol^−1^ at both 0 and 298 K regarding the initial elementary reactions undergone by xylose, 2-*O*-acetyl-xylose, and 4-*O*-methyl-d-glucuronic acid at different levels of theory

Elementary reaction	*T* = 0 K			*T* = 298 K		
	Δ*H*^‡^_DFT_	Δ*H*^‡^_CBS_	Δ*H*^‡^_G4_	Δ*H*^‡^_DFT_	Δ*H*^‡^_CBS_	Δ*H*^‡^_G4_
Xylose → d-xylose	49.68	38.19	45.55	45.54	37.30	45.54
Xylose → FF1 + H_2_O	76.21	71.20^a^	71.41	72.84	71.30^a^	71.47
Xylose → FF2 + H_2_O	73.19	57.55	65.07	70.25	56.68	65.09
Xylose → AXP1 + H_2_O	75.55	62.02	69.11	70.56	61.64	69.65
Xylose → AXP2 + H_2_O	87.20	73.70	80.73	82.00	73.27	81.08
Xylose → AXP3 + H_2_O	79.73	66.27	73.49	74.55	65.69	73.76
Xylose → AXP4 + H_2_O	81.45	67.78	74.86	76.08	67.40	75.20
Xylose → AXP5 + H_2_O	80.08	66.41	74.05	74.78	65.96	74.39
Xylose → AXP6 + H_2_O	81.68	67.01	74.38	76.07	66.46	74.85
Xylose → AXP7 + H_2_O	77.19	64.26	71.49	71.81	63.48	71.56
2-*O*-Acetyl-xylose → OADXP	46.56	43.99	43.91	42.65	43.89	43.79
2-*O*-Acetyl-xylose → FFL1 + CH_3_COOH	—	—	—	—	—	—
2-*O*-Acetyl-xylose → FFL2 + CH_3_COOH	71.64^a^	65.49	65.27	68.44^a^	65.61	65.35
2-*O*-Acetyl-xylose → AXPL1 + H_2_O	70.71	68.04	67.72	66.07	68.33	67.94
2-*O*-Acetyl-xylose → AXPL2 + CH_3_COOH	79.37	73.76	73.28	74.39	74.32	73.80
2-*O*-Acetyl-xylose → AXPL3 + CH_3_COOH	70.94	65.25	65.14	65.77	65.55	65.43
2-*O*-Acetyl-xylose → AXPL4 + H_2_O	75.77	72.43	71.94	70.84	72.72	72.19
2-*O*-Acetyl-xylose → AXPL5 + H_2_O	77.76	71.49	71.32	72.57	71.63	71.43
2-*O*-Acetyl-xylose → AXPL6 + H_2_O	76.33	73.10	72.38	71.16	73.58	72.79
2-*O*-Acetyl-xylose → AXPL7 + H_2_O	75.25	70.38	69.78	69.60	70.49	69.87
4-*O*-Methyl-d-glucuronic acid → DGLA	51.87	47.77	47.66	47.36	47.76	47.62
4-*O*-Methyl-d-glucuronic acid → FFL1 + H_2_O	82.85	76.77^a^	76.70^a^	79.21	77.02^a^	76.99^a^
4-*O*-Methyl-d-glucuronic acid → FFL2 + H_2_O	67.34	60.91	61.01	64.66	60.94	61.03
4-*O*-Methyl-d-glucuronic acid → AXPL1 + H_2_O	75.64	70.19	68.79	70.49	70.62	69.27
4-*O*-Methyl-d-glucuronic acid → AXPL2 + H_2_O	85.70	75.67	75.28	81.37	75.53	75.10
4-*O*-Methyl-d-glucuronic acid → AXPL3 + H_2_O	80.77	74.82	74.29	75.62	75.19	74.60
4-*O*-Methyl-d-glucuronic acid → AXPL4 + H_2_O	73.24	69.40	68.89	68.71	69.38	68.78
4-*O*-Methyl-d-glucuronic acid → AXPL5 + H_2_O	78.65	73.87	73.51	73.68	74.23	73.84
4-*O*-Methyl-d-glucuronic acid → AXPL6 + CH_3_OH	79.67	72.23	72.28	74.73	72.85	72.92
4-*O*-Methyl-d-glucuronic acid → AXPL7 + CH_3_OH	66.35	63.95	63.53	62.58	64.08	63.70

a Indicates relaxed convergence criteria.

It is worth noting that the transition states (TSs) of reactions comprising the conversion from the pyran to FF configuration, TS-FFL1, failed to converge for 2-*O*-acetyl-xylose at the DFT level regardless of utilizing Gaussian 16 algorithms such as synchronous transit-guided quasi-Newton (STQN) and scan. Attempts to explore the PES using the nudged elastic band (NEB) and NEB-TS algorithms implemented in ORCA 5.0^70^ proved unsuccessful in providing a reliable guess for this TS. In this context, the TS-FFL2 was finally located upon relaxing the convergence criteria by adjusting the Gaussian ConvF threshold, *i.e.*, ConvF = *N* × 10^−6^, where *N* = 3.0 × 10^5^. This approach was also adopted for xylose (CBS-QB3) and 4-*O*-methyl-d-glucuronic acid (CBS-QB3 and G4), using *N* = 1.2 × 10^4^ and *N* = 3.0 × 10^5^, respectively. These values have been indicated by an asterisk (see Table 1) and are excluded from our analysis.

Interestingly, the barrier description provided by G4 and CBS-QB3 differs substantially across functionalized and unfunctionalized species. This proves fundamental differences in the state-of-the-art levels of theory integrated within these composite models, particularly in their capabilities to describe correlation effects. The electronic energy accuracy provided by CCSD(T) and QCSD(T) plays a significant role in this discrepancy. In the case of the single-functionalized species, CBS-QB3 yields a significant difference of 
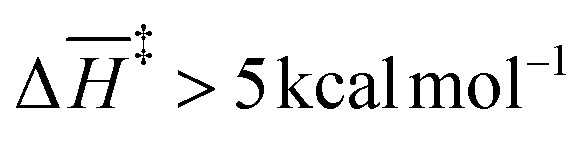
 for most steps relative to the simplest system. Contrary to the results obtained with G4. This Gaussian-family method reveals that the presence of the *O*-acetyl group at C2 significantly lowers the activation enthalpy (≈7–8 kcal mol^−1^) of processes directly involving the substituent, specifically TS-AXPL2 and TS-AXPL3. Similarly, this trend is observed when comparing xylose and 4-*O*-methyl-d-glucuronic acid results, as evidenced by the relatively high difference in Δ*H*^‡^ for the elimination comprising the methoxy group (undergone *via* TS-AXPL7). This is expected for TSs involving such a group; however, we found other dehydrations where Δ*H*^‡^ > 5 kcal mol^−1^ at the G4 level, exemplified by the reactions associated with TS-AXPL2 and TS-AXPL4. Therefore, conducting an in-depth examination of the steric and regioselectivity effects is pertinent to elucidate the underlying mechanisms influencing the apparent favorable direction.

The standard enthalpy of formation, Δ*H*^0^_f_(298 K), of atoms and molecules is an essential thermodynamic quantity in the analysis of gas-phase processes employing detailed kinetic models. This quantity is determined through different methodologies, including isodesmotic^71,72^ and connectivity-based hierarchy (CBH)^44,73^ schemes. In our study, we determined Δ*H*^0^_f_(298 K) for reactants and products resulting from the initial elementary steps through the atomization scheme outlined by Rogers and collaborators.^45^ This procedure combines experimental^46,47^ and atomization thermochemistry data generated *via* computational algorithms to calculate Δ*H*^0^_f_ by considering the reaction enthalpy of various isodesmic^74^ and homodesmotic^74^ reactions. Although less prone to error cancellations, the atomization approach was adopted due to its easy applicability and reasonable accuracy.^45,75^ The evaluation of water, methanol, and acetic acid (AA) in the gas phase reveals discrepancies not surpassing 0.50 kcal mol^−1^ between the experimental and computed values when corrections for spin–orbit couplings (SOC)^76,77^ of carbon and oxygen atoms are integrated *a posteriori* within the atomization scheme to fine-tune Δ*H*^0^_f_, as presented in Table 2. Notably, the absolute error decreases by 0.16, 0.20, and 0.50 kcal mol^−1^ for the corresponding species when such a correction is considered; these values are presented in parentheses in Table 2. This trend indicates the potential significant influence of SOC effects on the accuracy of the predicted Δ*H*^0^_f_, particularly for moieties characterized by a relatively high molecular weight.

**Table 2:** Standard enthalpy of formation, Δ*H*^0^_f_(298K), in kcal mol^-1^ of reactants and products resulting from ring-opening, ring-contraction, and eliminations. The values within parentheses exclude spin–orbit coupling corrections. The enthalpy of chemical species was computed at the G4 level

Species	Δ*H*^0^_f_	Formula	Species	Δ*H*^0^_f_	Formula	Species	Δ*H*^0^_f_	Formula
Xylose	−218.15 (−216.91)	C_5_H_10_O_5_	2-*O*-Acetyl-xylose	−263.08 (−261.51)	C_7_H_12_O_6_	4OMGLA	−299.99 (−298.26)	C_7_H_12_O_7_

DXP	−209.65 (−208.41)	C_5_H_10_O_5_	OADXP	−253.62 (−252.05)	C_7_H_12_O_6_	DGLA	−291.27 (−289.54)	C_7_H_12_O_7_
	
FF1	−145.27 (−144.20)	C_5_H_8_O_4_	FFL1	−145.28 (−144.20)	C_5_H_8_O_4_	FFL1	−229.11 (−227.55)	C_7_H_10_O_6_
FF2	−145.96 (−144.88)	C_5_H_8_O_4_	FFL2	−146.06 (−144.99)	C_5_H_8_O_4_	FFL2	−230.49 (−228.92)	C_7_H_10_O_6_

AXP1	−145.78 (−144.71)	C_5_H_8_O_4_	AXPL1	−189.18 (−187.77)	C_7_H_10_O_5_	AXPL1	−226.61 (−225.05)	C_6_H_8_O_6_
AXP2	−152.76 (−151.69)	C_5_H_8_O_4_	AXPL2	−152.77 (−151.69)	C_7_H_10_O_5_	AXPL2	−232.26 (−230.69)	C_6_H_8_O_6_
AXP3	−150.87 (−149.80)	C_5_H_8_O_4_	AXPL3	−148.49 (−147.41)	C_7_H_10_O_5_	AXPL3	−233.14 (−231.57)	C_7_H_10_O_6_
AXP4	−149.52 (−148.44)	C_5_H_8_O_4_	AXPL4	−190.48 (−189.08)	C_7_H_10_O_5_	AXPL4	−228.63 (−227.07)	C_7_H_10_O_6_
AXP5	−152.56 (−151.49)	C_5_H_8_O_4_	AXPL5	−199.94 (−198.54)	C_5_H_8_O_4_	AXPL5	−233.80 (−232.23)	C_7_H_10_O_6_
AXP6	−150.47 (−149.40)	C_5_H_8_O_4_	AXPL6	−200.34 (−198.93)	C_5_H_8_O_4_	AXPL6	−238.97 (−237.49)	C_7_H_10_O_6_
AXP7	−149.79 (−148.72)	C_5_H_8_O_4_	AXPL7	−202.59 (−201.19)	C_7_H_10_O_5_	AXPL7	−237.89 (−236.41)	C_7_H_10_O_6_
H_2_O	−57.46^a^ (−57.30)	H_2_O	AA	−102.95^b^ (−102.46)	C_2_H_4_O_2_	Methanol	−48.12^c^ (−47.87)	CH_4_O

a The experimental value is equal to (−57.80 ± 0.02) kcal mol^−1^. See Ruscic *et al.*^46^

b The experimental value is equal to (−103.41 ± 0.36) kcal mol^−1^. See Ruscic *et al.*^46^

c The experimental value is equal to (−48.07 ± 0.15) kcal mol^−1^. See Ruscic *et al.*^46^

The heat capacity of materials is a relevant quantity in thermodynamics, aiding in the precise calculation of thermal requirements and optimal processing temperatures. Furthermore, it serves as a discriminative parameter in distinguishing between two distinct polymeric composites based on their thermal characteristics.^78^ In this context, the heat capacities at constant volume (*C*_v_) and pressure (*C*_p_), as well as the entropy, were computed for all the species in the gas phase. The low values of *C*_p_ increase with rising temperatures for all species. As anticipated, the pyran-ring structures have lower values than their corresponding acyclic form since the latter molecular configuration is less rigid, which favors the contribution of additional vibrational degrees of freedom. The influence of functional groups is evident since larger structures, in terms of the number of atoms, are characterized by higher *C*_p_. For instance, at 200 K and 1.0 atm, the heat capacity of 2-*O*-acetyl-xylose and 4-*O*-methyl-d-gluguronic acid are 22.9 and 27.7% higher, respectively, than that of xylose. Such percentages slightly decrease as the absolute temperature increases.

At a temperature of 381.20 K and under 1.0 atm of pressure, calorimetric experiments conducted by McCullough and co-workers^79^ give a value of 0.0088 kcal mol^−1^ K^−1^ for the water vapor, characterized by an expected uncertainty not greater than ±0.2%. Notably, our calculations yield 0.0082 kcal mol^−1^ K^−1^ at 400 K, showing a close agreement with the experimental data. However, they applied statistical procedures to compensate for deviations driven by gas imperfections since the apparent heat capacity decreased as the temperature increased. This observation contrasts with our computational findings, which do not display such a trend, as evidenced in Table 3.

**Table 3:** Heat capacity, *C*_p_, in kcal mol^−1^ K^−1^ at different temperatures of chemical species involved in the initial elementary reactions: ring-opening, ring-contraction, and elimination. The dataset was generated using the frequency of normal modes calculated at M06-2X/6-311++G(d,p)

Species	*T*/K 200	300	400	500	600	700	800	900	1000
Xylose	0.02935	0.04030	0.05075	0.05989	0.06740	0.07349	0.07851	0.08270	0.08626
DXP	0.03244	0.04277	0.05263	0.06126	0.06839	0.07421	0.07904	0.08310	0.08657
FF1	0.02567	0.03435	0.04308	0.05081	0.05721	0.06245	0.06677	0.07038	0.07345
FF2	0.02591	0.03446	0.04312	0.05082	0.05722	0.06245	0.06678	0.07040	0.07346
AXP1	0.02544	0.03494	0.04385	0.05153	0.05780	0.06287	0.06704	0.07053	0.07348
AXP2	0.02539	0.03492	0.04389	0.05160	0.05787	0.06294	0.06709	0.07057	0.07351
AXP3	0.02481	0.03445	0.04352	0.05130	0.05763	0.06276	0.06696	0.07047	0.07345
AXP4	0.02507	0.03464	0.04364	0.05137	0.05768	0.06280	0.06699	0.07050	0.07346
AXP5	0.02495	0.03463	0.04365	0.05139	0.05770	0.06281	0.06701	0.07051	0.07348
AXP6	0.02531	0.03481	0.04376	0.05146	0.05776	0.06286	0.06705	0.07054	0.07351
AXP7	0.02526	0.03480	0.04385	0.05161	0.05792	0.06300	0.06716	0.07063	0.07357
H_2_O	0.00795	0.00800	0.00816	0.00839	0.00864	0.00890	0.00916	0.00943	0.00969
		(0.00803)	(0.00819)	(0.00842)	(0.00868)	(0.00895)	(0.00925)	(0.00955)	(0.00985)
2-*O*-Acetyl-xylose	0.03806	0.05136	0.06441	0.07588	0.08534	0.09305	0.09942	0.10475	0.10927
OADXP	0.04135	0.05416	0.06649	0.07735	0.08639	0.09383	0.10001	0.10522	0.10966
FFL1	0.02556	0.03428	0.04303	0.05078	0.05719	0.06243	0.06676	0.07038	0.07344
FFL2	0.02578	0.03441	0.04314	0.05088	0.05727	0.06250	0.06681	0.07042	0.07348
AXPL1	0.03416	0.04604	0.05748	0.06746	0.07569	0.08241	0.08796	0.09261	0.09654
AXPL2	0.02537	0.03491	0.04389	0.05159	0.05787	0.06294	0.06710	0.07057	0.07351
AXPL3	0.02469	0.03442	0.04358	0.05139	0.05772	0.06283	0.06701	0.07050	0.07345
AXPL4	0.03437	0.04607	0.05747	0.06745	0.07568	0.08242	0.08797	0.09262	0.09656
AXPL5	0.03388	0.04578	0.05732	0.06736	0.07562	0.08235	0.08791	0.09256	0.09650
AXPL6	0.03345	0.04548	0.05713	0.06723	0.07552	0.08229	0.08786	0.09252	0.09646
AXPL7	0.03257	0.04490	0.05683	0.06711	0.07549	0.08228	0.08786	0.09251	0.09644
AA	0.01268	0.01625	0.01977	0.02286	0.02546	0.02763	0.02946	0.03103	0.03239
		(0.01597)	(0.01952)	(0.02260)	(0.02515)	(0.02735)	(0.02908)	(0.03060)	(0.03199)
4OMGLA	0.04061	0.05469	0.06822	0.08008	0.08986	0.09783	0.10439	0.10987	0.11450
DGLA	0.04207	0.05623	0.06953	0.08107	0.09059	0.09837	0.10480	0.11019	0.11477
FFL1	0.03624	0.04838	0.06033	0.07085	0.07957	0.08671	0.09259	0.09750	0.10165
FFL2	0.03648	0.04849	0.06037	0.07086	0.07958	0.08671	0.09260	0.09752	0.10167
AXPL1	0.03629	0.04916	0.06124	0.07167	0.08023	0.08719	0.09291	0.09768	0.10171
AXPL2	0.03645	0.04930	0.06141	0.07185	0.08039	0.08733	0.09303	0.09778	0.10179
AXPL3	0.03556	0.04855	0.06081	0.07137	0.08001	0.08703	0.09279	0.09760	0.10165
AXPL4	0.03662	0.04928	0.06130	0.07172	0.08028	0.08724	0.09297	0.09774	0.10177
AXPL5	0.03580	0.04882	0.06102	0.07153	0.08014	0.08713	0.09286	0.09765	0.10168
AXPL6	0.03234	0.04440	0.05525	0.06432	0.07159	0.07740	0.08212	0.08601	0.08929
AXPL7	0.03308	0.04491	0.05559	0.06456	0.07177	0.07754	0.08221	0.08608	0.08934
Methanol	0.00946	0.01079	0.01270	0.01470	0.01653	0.01815	0.01956	0.02081	0.02191
		(0.01052)	(0.01229)	(0.01422)	(0.01602)	(0.01762)	(0.01904)	(0.02029)	(0.02138)

In a separate study, Stull and collaborators^80^ compiled a thermochemical dataset spanning over several decades, comprising 741 pure organic compounds in the gas phase. This type of collection of experimental values was previously obtained by combining thermal and spectroscopic direct measurements. For water, the computed *C*_p_ in the range of 300 to 1000 K deviates by 1.6% at most from the corresponding experimental value. Furthermore, this remarkable trend is also observed for acetic acid (AA) and methanol; see Table 3.

Goldberg and collaborators^81^ performed rigorous combustion calorimetric measurements on the crystalline alpha anomer of xylose, specifically α-d-xylopyranose. Their investigation covered a wide temperature range from 1.9 to 305 K and used an isoperibol static bomb calorimeter. They reported an experimental value equal to *C*_p_ = (0.04254 ± 0.00180) kcal mol^−1^ K^−1^ at 298.15 K, which deviates 3.21% from other measures obtained through similar experimental setups.^82,83^ Surprisingly, the *C*_p_ provided by Goldberg closely approximates the computed one for the beta isomer in the gas phase at 300 K. In contrast, the *C*_p_ = (0.06716 ± 0.002) kcal mol^−1^ K^−1^ of the *α* configuration in the aqueous phase at 303 K found by Kawaizumi *et al*.^83^ is about 60% higher. Additional details concerning computed values of *C*_v_ and *S* for all species in the gas phase can be found in the ESI.†

**Fig. 1:**
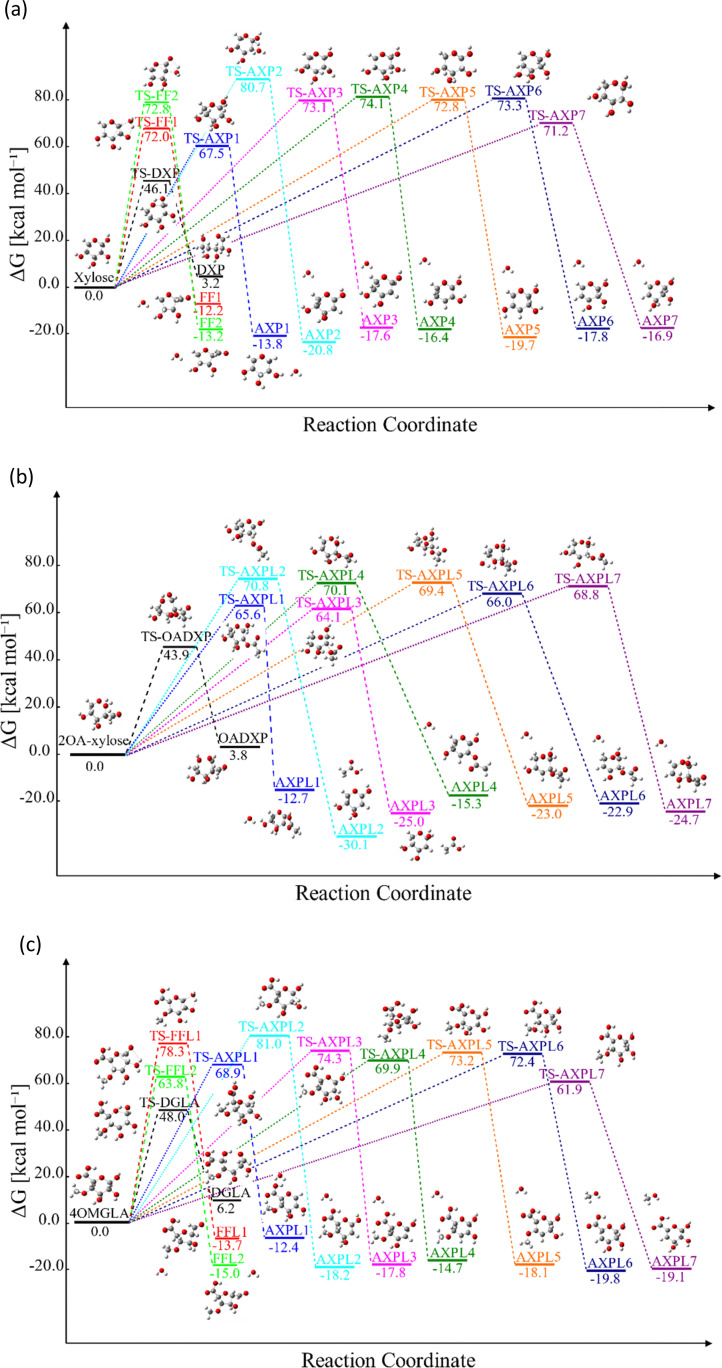
Gibbs free energy, Δ*G*, at 773.15 K of key structures featuring the thermal depolymerization of hemicellulose structural motifs: xylose (panel a), 2-*O*-acetyl-xylose (panel b), and 4-*O*-methyl-d-glucuronic acid (panel c). DXP, OADXP, and DGLA denote the acyclic fragments of the ring-opening of xylose, 2-*O*-acetyl-xylose, and 4-*O*-methyl-d-glucuronic acid, respectively. FF*j* and FFL*j* (*j* = 1,2) correspond to the products of the ring-contraction, and AXP*i* and AXPL*i* (*i* = 1, 2,…,7) represent the moieties resulting from eliminations undergone by xylose and functionalized species, respectively. The molecular configurations are color-coded: carbons in grey, oxygens in red, and hydrogens in white. Δ*G* was obtained by gauging the frequency of normal modes computed at M06-2X/6-311++G(d,p).

Reactive potential energy surfaces of the three investigated systems are reported in Fig. 1.

### Kinetic findings

The analysis of the Gibbs free energy, Δ*G*, reveals distinct thermodynamic behaviors among the species studied. For xylose, the ring-opening step becomes favorable only at temperatures exceeding 800 K. In contrast, Δ*G* > 0 for both substituted systems in the temperature interval of 400 to 1000 K indicates the thermodynamic non-favorability of this reaction. All the other processes proceed in the forward direction across this temperature range for the simplest reactant and 4-*O*-methyl-d-glucuronic acid. However, the elimination process leading to the AXPL1 product is unfavorable at the lowest temperature for the single-substituted system, as shown in Table 4. It is worth noting that 400 K falls completely outside the FP domain.

**Table 4:** Gibbs free energies, Δ*G*, in kcal mol^−1^ of reactants and products resulting from the ring-opening, ring-contraction, and elimination initial steps. The data was generated by leveraging the frequency of normal modes computed at M06-2X/6-311++G(d,p)

Reaction	*T*/K 400	500	600	700	800	900	1000
Xylose	0	0	0	0	0	0	0
d-Xylose	2.01	1.49	0.99	0.49	0.01	−0.45	−0.90
FF1 + H_2_O	−1.72	−5.80	−9.84	−13.84	−17.79	−21.70	−25.56
FF2 + H_2_O	−2.71	−6.82	−10.89	−14.91	−18.89	−22.82	−26.70
AXP1 + H_2_O	−0.87	−4.83	−8.79	−12.74	−16.66	−20.56	−24.43
AXP2 + H_2_O	−7.83	−11.84	−15.85	−19.86	−23.83	−27.79	−31.71
AXP3 + H_2_O	−5.72	−9.58	−13.44	−17.27	−21.08	−24.85	−28.60
AXP4 + H_2_O	−4.26	−8.16	−12.05	−15.93	−19.77	−23.59	−27.38
AXP5 + H_2_O	−6.67	−10.60	−14.53	−18.44	−22.33	−26.20	−30.03
AXP6 + H_2_O	−5.13	−9.06	−12.99	−16.89	−20.77	−24.63	−28.45
AXP7 + H_2_O	−3.05	−7.05	−11.04	−15.01	−18.96	−22.88	−26.77
2-*O*-Acetyl-xylose	0	0	0	0	0	0	0
OADXP	4.32	3.73	3.13	2.53	1.94	1.34	0.75
FFL1 + CH_3_COOH	−3.89	−8.82	−13.74	−18.65	−23.55	−28.42	−33.29
FFL2 + CH_3_COOH	−5.06	−9.99	−14.92	−19.84	−24.74	−29.62	−34.49
AXPL1 + H_2_O	1.43	−2.58	−6.60	−10.61	−14.59	−18.56	−22.50
AXPL2 + CH_3_COOH	−15.11	−19.92	−24.75	−29.60	−34.44	−39.28	−44.11
AXPL3 + CH_3_COOH	−11.49	−16.21	−20.94	−25.67	−30.38	−35.08	−39.76
AXPL4 + H_2_O	−0.73	−4.87	−9.01	−13.14	−17.25	−21.34	−25.40
AXPL5 + H_2_O	−9.44	−13.42	−17.40	−21.36	−25.30	−29.22	−33.10
AXPL6 + H_2_O	−9.64	−13.56	−17.47	−21.38	−25.27	−29.12	−32.95
AXPL7 + H_2_O	−10.00	−13.84	−17.68	−21.52	−25.35	−29.15	−32.93
4OMGLA	0	0	0	0	0	0	0
DGLA	2.19	1.98	1.77	1.57	1.36	1.17	0.97
FFL1 + H_2_O	−4.34	−8.41	−12.46	−16.46	−20.43	−24.35	−28.24
FFL2 + H_2_O	−5.81	−9.92	−14.00	−18.04	−22.04	−26.00	−29.92
AXPL1 + H_2_O	−0.05	−4.02	−7.99	−11.95	−15.89	−19.80	−23.68
AXPL2 + H_2_O	−5.23	−9.30	−13.37	−17.44	−21.49	−25.52	−29.51
AXPL3 + H_2_O	−6.76	−10.72	−14.68	−18.62	−22.54	−26.43	−30.29
AXPL4 + H_2_O	−2.52	−6.62	−10.71	−14.78	−18.83	−22.86	−26.85
AXPL5 + H_2_O	−5.58	−9.58	−13.58	−17.58	−21.56	−25.52	−29.45
AXPL6 + CH_3_OH	−7.34	−11.88	−16.41	−20.93	−25.44	−29.92	−34.38
AXPL7 + CH_3_OH	−6.92	−11.58	−16.25	−20.91	−25.55	−30.17	−34.77

**Fig. 2:**
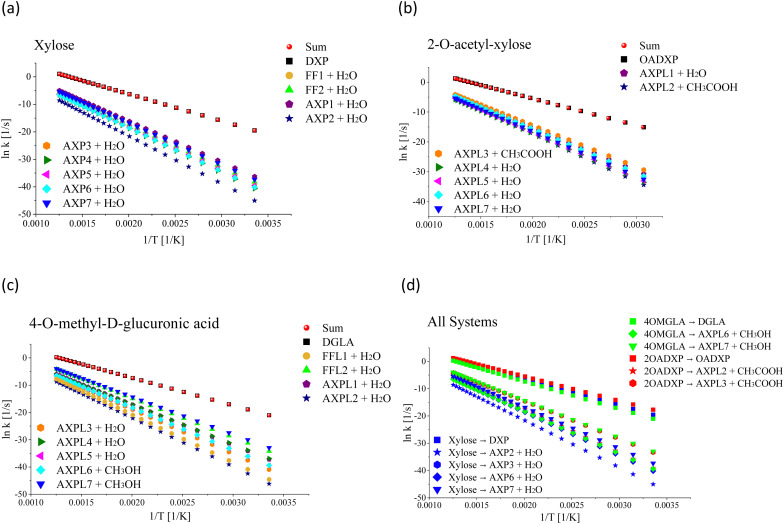
Rate constant dependence on the inverse of the absolute temperature. Panel (a) shows the rate constant of the initial decomposition reaction of xylose: ring-opening (xylose → DXP), ring-contractions (xylose → FF1 + H_2_O and xylose → FF2 + H_2_O), and eliminations (Xylose → AXP*i* + H_2_O, *i* = 1,…,7). Panel (b) depicts the rate constant of the initial decomposition of 2-*O*-acetyl-xylose: ring-opening (2-*O*-acetyl-xylose → OADXP) and eliminations (2-*O*-acetyl-xylose → AXPL*j* + H_2_O, *j* = 1, 4,…,7 and 2-*O*-acetyl-xylose → AXPL*k* + CH_3_COOH, *k* = 2, 3). Panel (c) shows the rate constant of the initial decomposition of 4-*O*-methyl-d-glucuronic acid: ring-opening (4OMGLA → DGLA), ring-contractions (4OMGLA → FFL1 + H_2_O and 4OMGLA → FFL2 + H_2_O), and eliminations (4OMGLA → AXPL*l* + H_2_O, *l* = 1,…,5 and 4OMGLA → AXPL*m* + CH_3_COOH, *m* = 6, 7). Panel (d) stresses that each process is mostly influenced by the functional group active in the transition state geometry; however, the magnitude of this effect strongly depends on the side of the proton transfer. This is illustrated *via* color-coded symbols: xylose in red, 2-*O*-acetyl-xylose in blue, and 4-*O*-methyl-d-glucuronic acid in green. The dataset results from the statistical treatment of properties computed at M06-2X/6-311++G(d,p).

From a kinetic perspective, the ring-opening reaction exhibits the highest rate for all species; see Fig. 2, panels a–c. However, the presence of substituents induces some qualitative and quantitative changes. Among all processes, the ring-opening shows the least sensitivity to temperature changes, evidenced by the lowest tilt of its rate line. The functional groups close the competing gap as the temperature increases, which is more evident for the moiety characterized by the highest number of substituent groups, *i.e.*, 4-*O*-methyl-d-glucuronic acid. For xylose, the water elimination at C1 undergone through TS-AXP1, the dehydration at C4 associated with TS-AXP7, and the ring-contraction linked to TS-FF2 are the second, third, and fourth fastest processes, respectively; in contrast, this ranking shifts to TS-AXPL7, TS-FFL2, and TS-AXPL1 for the double-substituted species. In the case of 2-*O*-acetyl-xylose, one of the reactions involving the substituent (TS-AXPL3) will still lie within the three fastest depolymerization events, even assuming that both elementary reactions associated with TS-FFL1 and TS-FFL2 could be faster. Surprisingly, the other elimination involving the functional group (TS-AXPL2) is the lowest reaction. These kinetics further support our previous thermodynamic findings regarding the relevant role of proton-transfer directionality (see Fig. 2, panel d), thereby underscoring the need for an in-depth assessment of selectivity and steric effects. Regardless of the relatively high vibrational frequency of TSs associated with ring-opening and eliminations, the Eckart tunneling effects are extremely weak because temperatures characterizing the FP regimen greatly surpass the cross-over temperature (≈92–458 K). Consequently, the tunneling is shallow and points out that rate constants can be computed using the classical rate theory.^84,85^

Table 5 presents a detailed overview of the frequency factor (*A*) and activation energy (*E*_a_) values derived from fitting the thermal rate constant data depicted in Fig. 2, panels a–c, to a two-parameter Arrhenius model. In the case of the simplest system, the highest values of *A* correspond to the ring-contractions FF1 and FF2. In contrast, for the *O*-acetyl functionalized species, the *A* maximum value corresponds to one of the elementary reactions involving the substituent, *i.e.*, TS-AXPL2. As expected, the same trend is observed in the case of the double-functionalized system. This shows the consistency between the thermodynamic and kinetic findings that substituents indeed exert a catalytic-like effect in both functionalized species. However, the extent of this effect is contingent upon the relative spatial arrangement of the reacting centers, particularly the specific side of the proton attack. More importantly, while discernible, the influence of functional groups is insufficient to substantially bridge the competition gap between the ring-opening reaction and the other elementary steps within the FP regime.

**Table 5:** Arrhenius fit parameters for the initial reactions of substituted and unsubstituted systems over the interval [400, 1000] K. The rate constant, *k*, is computed at 773.15 K

Elementary reaction	*A* [s^−1^]	*E*_a_ [kcal mol^−1^]	*k* [s^−1^]	*k*/*k*_sum_ [%]
Xylose → d-xylose	1.68 × 10^13^	44.68	4.11	100.00
Xylose → FF1 + H_2_O	8.79 × 10^13^	72.71	2.63 × 10^−7^	6.40 × 10^−6^
Xylose → FF2 + H_2_O	7.44 × 10^13^	64.62	4.28 × 10^−5^	1.04 × 10^−3^
Xylose → AXP1 + H_2_O	3.79 × 10^13^	68.06	2.33 × 10^−6^	5.70 × 10^−5^
Xylose → AXP2 + H_2_O	1.17 × 10^13^	79.31	4.80 × 10^−10^	1.17 × 10^−8^
Xylose → AXP3 + H_2_O	3.17 × 10^13^	72.39	1.17 × 10^−7^	2.85 × 10^−6^
Xylose → AXP4 + H_2_O	5.06 × 10^13^	74.17	5.86 × 10^−8^	1.42 × 10^−6^
Xylose → AXP5 + H_2_O	2.46 × 10^13^	72.26	9.86 × 10^−8^	2.40 × 10^−6^
Xylose → AXP6 + H_2_O	3.26 × 10^13^	73.28	6.74 × 10^−8^	1.64 × 10^−6^
Xylose → AXP7 + H_2_O	1.69 × 10^13^	70.12	2.72 × 10^−7^	6.62 × 10^−6^
2-*O*-Acetyl-xylose → OADXP	3.23 × 10^12^	41.87	4.90	100.00
2-*O*-Acetyl-xylose → FFL1 + CH_3_COOH	—	—	—	—
2-*O*-Acetyl-xylose → FFL2 + CH_3_COOH	—	—	—	—
2-*O*-Acetyl-xylose → AXPL1 + H_2_O	3.95 × 10^13^	66.27	7.77 × 10^−6^	1.58 × 10^−4^
2-*O*-Acetyl-xylose → AXPL2 + CH_3_COOH	5.03 × 10^14^	73.31	1.02 × 10^−6^	2.08 × 10^−5^
2-*O*-Acetyl-xylose → AXPL3 + CH_3_COOH	1.84 × 10^13^	63.64	2.00 × 10^−5^	4.08 × 10^−4^
2-*O*-Acetyl-xylose → AXPL4 + H_2_O	3.94 × 10^13^	69.62	8.78 × 10^−7^	1.79 × 10^−5^
2-*O*-Acetyl-xylose → AXPL5 + H_2_O	8.61 × 10^13^	70.33	1.21 × 10^−6^	2.47 × 10^−5^
2-*O*-Acetyl-xylose → AXPL6 + H_2_O	2.43 × 10^13^	68.66	1.01 × 10^−6^	2.06 × 10^−5^
2-*O*-Acetyl-xylose → AXPL7 + H_2_O	3.97 × 10^13^	69.62	8.85 × 10^−7^	1.81 × 10^−5^
4-*O*-Methyl-d-glucuronic acid → DGLA	8.39 × 10^12^	46.41	6.66 × 10^−1^	99.95
4-*O*-Methyl-d-glucuronic acid → FFL1 + H_2_O	1.45 × 10^14^	80.14	3.48 × 10^−9^	5.22 × 10^−7^
4-*O*-Methyl-d-glucuronic acid → FFL2 + H_2_O	1.23 × 10^13^	62.20	3.41 × 10^−5^	5.12 × 10^−3^
4-*O*-Methyl-d-glucuronic acid → AXPL1 + H_2_O	3.38 × 10^12^	67.94	2.25 × 10^−7^	3.38 × 10^−5^
4-O-Methyl-d-glucuronic acid → AXPL2 + H_2_O	1.77 × 10^12^	76.56	4.34 × 10^−10^	6.51 × 10^−8^
4-*O*-Methyl-d-glucuronic acid → AXPL3 + H_2_O	5.39 × 10^13^	74.64	4.60 × 10^−8^	6.90 × 10^−6^
4-*O*-Methyl-d-glucuronic acid → AXPL4 + H_2_O	1.19 × 10^12^	66.99	1.47 × 10^−7^	2.21 × 10^−5^
4-*O*-Methyl-d-glucuronic acid → AXPL5 + H_2_O	8.93 × 10^13^	71.50	5.87 × 10^−7^	8.81 × 10^−5^
4-*O*-Methyl-d-glucuronic acid → AXPL6 + CH_3_OH	1.04 × 10^13^	71.53	6.70 × 10^−8^	1.01 × 10^−5^
4-*O*-Methyl-d-glucuronic acid → AXPL7 + CH_3_OH	1.80 × 10^14^	63.04	2.89 × 10^−4^	4.34 × 10^−2^

### Chemical reactivity and bonding analysis

Macro thermodynamic variables, such as heat capacity, originate from micro-level features. The kinetics of particles (*e.g.*, vibrational and translational velocities) serve as the physical basis of an intensive state variable we call temperature. This emphasizes the significance of delving into the molecular physics underpinning the thermal conversion of biomass into a wide variety of bioproducts as a critical step before integrating sustainable materials into practical applications. The valence-shell electron-pair repulsion theory (VSEPR)^86^ leverages electrostatic considerations to accurately predict the molecular geometry of a wide variety of systems ranging from organic to inorganic compounds. Given the fundamental relationship between geometry, properties, and applications, our approach involves computing the electron population of each reactant as an initial step. This calculation constitutes a building block for establishing the intricate link between macro-level thermodynamics and micro-level molecular interactions through chemical bonding analysis of ELF. This kind of methodical approach has been broadly applied in both the solid-state and gas-phase to deepen our understanding across different covalent bonding situations,^87,88^ proton transfer reactions,^89^ C–H bonds activation,^90^ phase transition of the 14 group elements,^91^ cycloadditions,^92–99^ hemiaminals formation,^100,101^ photoreactions,^99,102,103^ arynes production,^104^ computing local and global properties of solids,^105^ and deducing linear models for predicting activation enthalpies of organic and organometallic reacting systems.^106^

As expected, ELF disynaptic basins associated with the substituent positions show the most substantial variations in population (≈0.1 e), specifically V(C2,O2) and V(C4,O4). Nonetheless, the *O*-acetyl group exerts a discernible influence on nearby basins, as evidenced by the decrease of approximately 0.1 e in the V(C1,O1) population. While electron populations along the pyran ring maintain near-constant levels, the most relevant changes (±0.03 e) are observed over V(C1,O), the bonding region featuring the ring-opening process. The relatively low population of V(C1,O), V(C1,O1), V(C2,O2), V(C3,O3), and V(C4,O4) provides insights into the significance attributed to ring opening/contraction and eliminations as these classes of reactions comprise such basins, as detailed in Table 6.

**Table 6:** Electron population (e) of xylose, 2-*O*-acetyl-xylose, and 4-*O*-methyl-d-glucuronic acid at the M06-2X/6-311++G(d,p) level

ELF basin^a^	Xylose	2-*O*-Acetyl-xylose	4-*O*-Methyl-d-glucuronic acid
V(C1,O)	1.35	1.38	1.33
V(C1,C2)	2.08	2.08	2.07
V(C2,C3)	2.02	2.01	2.02
V(C3,C4)	2.02	2.02	2.02
V(C4,C5)	2.01	2.01	1.99
V(C5,O)	1.29	1.29	1.29
V(C1,O1)	1.41	1.36	1.42
V(C2,O2)	1.29	1.39	1.29
V(C3,O3)	1.28	1.28	1.29
V(C4,O4)	1.27	1.27	1.32

a The labeling of oxygen matches the pattern delineated by the C*i*–O*i* bonding notation.

Both the global and local chemical reactivity indices^51,52,107^ leverage electron density as a foundational parameter to extract meaningful chemical information. This physical-observable-based approach streamlines the correlation of theoretical insights with experimental observations, thereby providing depth to the analysis in the context of thermokinetic discussions. Over recent decades, Fukui's functions have become a subject of increasing interest in both biomass materials and biomass pyrolysis fields, with diverse and impactful applications. López and co-workers^108^ identified the most reactive sites of two lignin precursors in reactions involving radicals. Benguerba *et al.*^109^ proposed agricultural olive cake waste as a low-cost material for absorbing acids. Muñiz and collaborators^110^ developed a methodology to predict the performance of carbon-based electrode materials for energy storage by assessing the reaction mechanism of various lignocellulosic components resulting from the pyrolysis of *Agave angustifolia* leaves. Yáñez *et al.*^111^ studied the influence of a solvent-water mixture used to extract lignin (from *Eucalyptus nitens*) on its structural features and content of functional groups. Very recently, Xia and co-workers^112^ prepared graft-modified fibbers from starch polymers as alternative adhesives.

The electronic chemical potential, *μ*, reveals that the *O*-acetyl functionalized species is the most reactive (−4.80 eV), whereas the double substituted reactant displays the lowest reactivity (−4.62 eV). In contrast, xylose's susceptibility to exchanging electron density falls between these two values (*i.e.*, *μ* = −4.75 eV). The relatively high global reactivity of 2-*O*-acetyl-xylose might be indicative of the moderate thermodynamic stability of the functional group since they can be straightforwardly dissociated through acetic acid, as observed in experiments.^27,30^

**Fig. 3:**
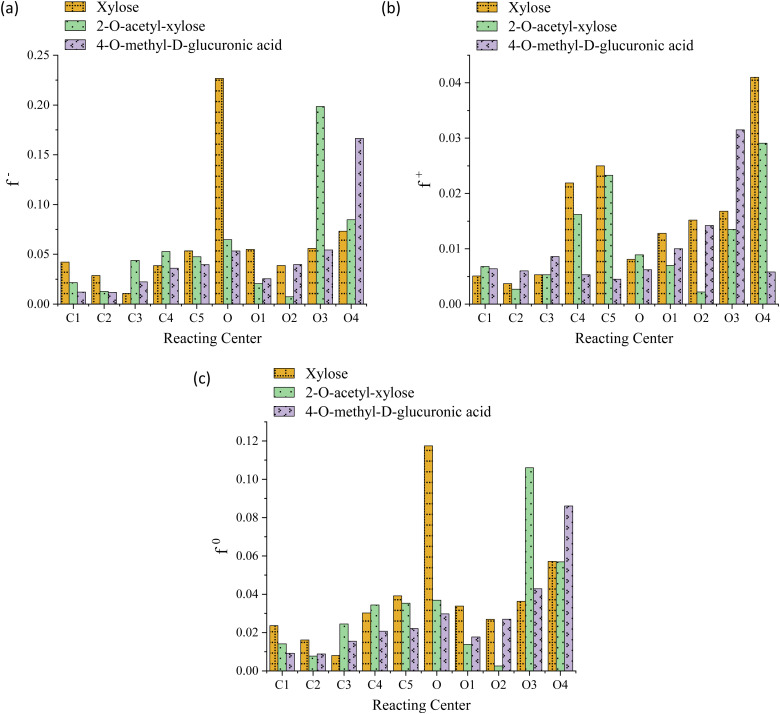
Fukui's functions *f*^*−*^ (panel a), *f*^+^ (panel b), and *f*^0^ (panel c) of reacting centers involved in ring opening/contraction and eliminations undergone by xylose, 2-*O*-acetyl-xylose, and 4-*O*-methyl-d-glucuronic acid reactants at the M06-2X/6-311++G(d,p) level. The labeling of centers matches the pattern delineated by the C*i*–O*i* bonding notation.

The striking low values of Fukui's functions *f*^+^, *f*^−^, and *f*^0^ reveal the extremely weak local reactivity of the investigated chemical species and justify the need for FP conditions. Moreover, this suggests the implausibility of the investigated processes being reversible, aligning with their thermodynamically favorable nature. Both the *O*-acetyl and methoxy functional groups consistently modulate the propensity of the system toward electrophilic attacks near C2 and C4, respectively; in contrast, while the latter significantly increases this probability at O4, the former lowers it in the locality of O2, as evidenced in Fig. 3, panel a. The value of *f*^+^ drops at the C2, C4, O2, and O4 centers, indicating that the substituents lower the propensity to a nucleophilic attack in their vicinity, see Fig. 3, panel b. The C2, C4, and O2 regions are less prone to a neutral (or radical) attack, while the probability notably increases at O4, see Fig. 3, panel c. For xylose, the preeminent reactivity of the pyran-ring oxygen might potentially support the notion that the ring-opening is the fastest reaction, as indicated by the highest values of *f*^−^ and *f*^0^. Similarly, 4-*O*-methyl-d-glucuronic acid exhibits peak values for these functions for the methoxy oxygen (O4), possibly justifying the barrier reduction and the relatively high rate observed in one of the processes involving this functional group, namely, the elimination associated with TS-AXPL7. Intriguingly, in the case of 2-*O*-acetyl-xylose, all Fukui's functions are minimized at both C2 and O2, whereas *f*^−^ and *f*^0^ are maximized at the C3 and O3 atoms. This signifies that *O*-acetyl diminishes the centers reactivity at the substituent position, while significantly favors it at the neighboring reacting center, C3. Nonetheless, these observations closely match the thermokinetic findings since the elimination undergone *via* TS-AXPL3 involving C3 is the reaction with the lowest barrier and highest rate, excluding ring-opening. For functionalized species, none of these local functions exhibit a notable peak at the pyran-ring oxygen, which contradicts the results derived from the thermokinetic analysis. Thus, these outcomes fall short of elucidating the thermokinetic observations across all structural motifs consistently.

Although Fig. 3 suggests that Fukui's functions might contribute to the learning of molecular thermodynamic properties of large and complex biomass systems, as demonstrated for other materials and processes, further work is needed. Therefore, forthcoming studies will focus on formulating this qualitative nexus into a predictive tool. A systematic assessment of alternative electron-density-based descriptors will be conducted to test their appropriateness. The integration of well-established theories, such as the Quantum Theory of Atoms in Molecules (QTAIM),^113^ will be considered as well.

## Conclusions

In this study, we have analyzed the thermodynamic and kinetic effects of functional groups on the pyrolytic depolymerization of β-d-xylopyranose, a hemicellulose motif. Two distinct moieties, namely, 2-*O*-acetyl-β-d-xylopyranose and 4-methoxy-5-carboxy-β-d-xylopyranose, have been carefully selected to conduct this investigation as they have been experimentally isolated from various types of hemicellulose plant matter, a biomass main component relatively less studied compared to cellulose. Through state-of-the-art density functionals, composite chemistry methods, and transition state theory, we here reported quite complete thermochemical data for these carbohydrates for the first time to aid in developing reliable kinetic models. This dataset includes activation barriers, standard enthalpies of formation, Gibbs free energies, heat capacities, and rate constants at various temperatures for three initial reaction classes understood to decompose carbohydrates under pyrolysis conditions: ring-opening, ring-contraction, and elimination. The results demonstrated high reliability, as evidenced by the close agreement (*i.e.*, below 0.5 kcal mol^−1^) between computed and experimental enthalpies of formation for pyrolytic products of elimination reactions: water, acetic acid, and methanol. In this vein, the predicted standard enthalpy of formation of β-d-xylopyranose, 4-methoxy-5-carboxy-β-d-xylopyranose, and 2-*O*-acetyl-β-d-xylopyranose are determined to be −218.2, −263.1, and −300.0 kcal mol^−1^, respectively. Across all examined species, the ring-opening barrier fell approximately within 43.8–47.5 kcal mol^−1^, in contrast to the higher activation enthalpies observed for ring-contraction and elimination, ranging from 61.0 to 81.1 kcal mol^−1^. Regardless of the functionalization, the pathway leading to acyclic products was the fastest, featuring a rate constant in the order of 10 s^−1^, significantly superior to the rate characterizing the other reaction types, which ranged from 10^−10^ and 10^−4^ s^−1^ for the other reaction types. Curiously, the most reactive elementary step is the only thermodynamically unfavorable reaction within the Fast Pyrolysis regime. The thermochemical and kinetic analyses consistently showed that introducing functional groups lowers the barrier by 1.9 to 8.3 kcal mol^−1^. Consequently, the rate constant increased by 0 to 4 orders of magnitude concerning the unfunctionalized system. However, this catalytic-like effect strongly depends on the relative spatial rearrangements of reacting centers and proved insufficient to effectively bridge the competition between processes, evidencing the significant role of steric effects and regioselectivity. In light of these quantitative outcomes, it seems reasonable that, although the transition states associated with the reaction channels leading to the decomposition of 2-*O*-acetyl-β-d-xylopyranose into five-membered ring structures could not be found, such processes are not expected to compete with the ring-opening, as the latter would still lay more than 10 kcal mol^−1^ below and be ten times kinetically faster in the most favorable scenario. Furthermore, our study underscores the importance of conformational analysis, which should be considered more frequently given the geometric flexibility observed in biomass main components. It is quite promising that a straightforward chemical reactivity and bonding analysis offer partial corroboration for the thermokinetic findings. These insights notably simplify the exploration of potential energy surfaces of hemicelluloses pyrolysis by offering to circumvent numerous alternative depolymerization pathways.

## Author contributions

L. A.-H., S. D.: project supervision, conceptualization, data analysis, and manuscript writing; L. A.-H., J. L.: calculations, reaction paths, and characterization. All authors reviewed and commented on the manuscript.

## Conflicts of interest

There are no conflicts to declare.

## Supplementary Material

CP-026-D3CP06094B-s001
