# Supplementary material for: Hemicellulose pyrolysis: mechanism and kinetics of functionalized xylopyranose
Source: Phys Chem Chem Phys. 2024 Apr 4;26(16):12820–37. doi: 10.1039/d3cp06094b (PMC11041873; doi:10.1039/d3cp06094b)
Supplement: CP-026-D3CP06094B-s001 [file CP-026-D3CP06094B-s001.pdf]

Electronic Supplementary Material (ESI) for Physical Chemistry Chemical Physics.
This journal is © the Owner Societies 2024

Electronic Supplementary Information

Hemicellulose Pyrolysis: Mechanism and Kinetics of Functionalized Xylopyranose

Leandro Ayarde-Henríquez,[*ab] Jacopo Lupi,[ab] and Stephen Dooley[*ab]

[a]School of Physics, Trinity College Dublin, Dublin 2, Ireland.
[b]AMBER, Advanced Materials and BioEngineering Research Centre, Dublin 2, Ireland.

Correspondence to: leandro.ayarde@tcd.ie
stephen.dooley@tcd.ie

Contents

1. Optimized Cartesian coordinates for minima and TSs of β-D-xylopyranose's initial reactions at the M06-2X/6-311++G(d,p) level S1

2. Optimized Cartesian coordinates for minima and TSs of 2-O-acetyl-β-D-xylopyranose's initial reactions at the M06-2X/6-311++G(d,p) level S13

3. Optimized Cartesian coordinates for minima and TSs of 4-methoxy-5-carboxy-β-D-xylopyranose's initial reactions at the M06-2X/6-311++G(d,p) level S25

Table S1. Heat capacity, *Cv*, in kcal mol^{-1} K^{-1} at different temperatures of chemical species involved in the initial elementary reactions: ring opening, ring contraction, and elimination. S41

Table S2. Entropy, *S*, in kcal mol^{-1} K^{-1} at different temperatures of chemical species involved in the initial elementary reactions: ring opening, ring contraction, and elimination. S42

Table S3. $\mathcal{T}_1$ diagnostic. S43

1. Optimized Cartesian coordinates for minima and TSs of β-D-xylopyranose's initial reactions at the M06-2X/6-311++G(d,p) level

<u>Ring-opening (β-D-xylopyranose ⟶ D-xylose)</u>

Reactant (β-D-xylopyranose)

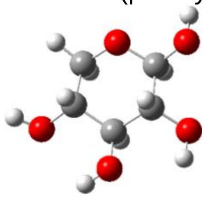

C	-1.38469600	-0.55335500	-0.29388900
C	-0.56258400	-1.72802300	0.23068100
C	1.43885800	-0.46884400	0.25359100
C	0.72491200	0.79435700	-0.20863900
C	-0.74134800	0.73030000	0.19135300
H	-0.60475900	-1.73503100	1.32941900

H	-0.94859500	-2.68003500	-0.13626100
H	-1.38478600	-0.56048900	-1.39048400
H	1.45194900	-0.50082900	1.35597300
H	0.80216800	0.83627300	-1.30345000
H	-0.80332500	0.74664400	1.29030900
O	0.76789200	-1.62214400	-0.23076700
O	2.71462500	-0.46820200	-0.27402300
H	3.16164700	-1.26936100	0.01422100
O	1.34772000	1.90356100	0.39097100
H	0.81365900	2.67634700	0.17899200
O	-1.36628300	1.87925700	-0.34293500
H	-2.29784300	1.84353800	-0.10300000
O	-2.70969800	-0.55411700	0.21411700
H	-3.23855900	-1.19964600	-0.26052300

Product (D-xylose)

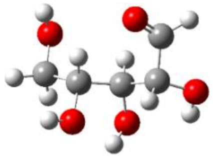

C	1.20797700	0.23390500	-0.34072700
C	2.16531900	1.00036100	0.56916900
C	-1.85741800	0.45722700	-1.04054500
C	-1.13937200	-0.75412700	-0.48107800
C	0.01792500	-0.31375500	0.44187800
H	2.42895100	0.37868700	1.42627100
H	3.08555400	1.23292900	0.01838500
H	0.83763000	0.89550600	-1.13185500
H	-2.87131700	0.61517200	-0.62640700
H	-0.72126400	-1.33416700	-1.31556300
H	-0.33842100	0.45085100	1.13737600
O	1.55863000	2.16798700	1.08315700
O	-1.37435100	1.20665800	-1.84677900
H	1.41172500	2.79011400	0.36547900
O	-2.08602000	-1.49795100	0.24266600
H	-1.59912300	-2.00492600	0.90373900
O	0.41133900	-1.42108800	1.23174400
H	1.03020100	-1.94184600	0.70258800
O	1.85034500	-0.90666800	-0.90156000
H	2.45895300	-0.63878500	-1.59321700

TS-DXP

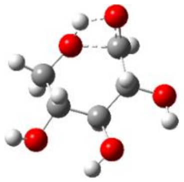

C	1.26257400	-0.53164900	0.31987800

C	0.65851400	-1.74916600	-0.37227000
C	-1.73006000	-0.25874800	-0.19854500
C	-0.81193600	0.89259700	0.21160200
C	0.63027700	0.73184700	-0.24707700
H	1.03808700	-1.78624100	-1.39664400
H	0.92870100	-2.67937000	0.13329100
H	1.09059900	-0.55898900	1.40330700
H	-2.14701100	-0.12944200	-1.20407100
H	-0.82544000	0.94458200	1.30750700
H	0.66057200	0.68403400	-1.34640400
O	-0.75983600	-1.66127000	-0.41683300
O	-2.42670000	-0.86946600	0.70876800
H	-1.52902000	-1.77869000	0.45303600
O	-1.37500000	2.05309500	-0.35611300
H	-0.77494000	2.78269800	-0.16582300
O	1.30200900	1.88644500	0.20843500
H	2.24458100	1.75331600	0.05922200
O	2.65129000	-0.44699200	0.03817600
H	3.14382200	-1.01763200	0.63238400

Ring contraction (β-D-xylopyranose ⟶ FF1 + H_2O)

Product (FF1)

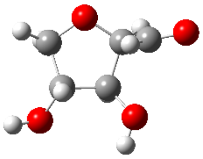

C	1.22930900	1.44415600	-0.02052700
C	1.36417100	-0.02805000	-0.38962800
C	0.28792200	-0.64704800	0.49326400
C	-0.80147400	0.44373600	0.51689800
C	-1.93076700	0.11611100	-0.44967900
H	1.08707200	-0.18461300	-1.43974200
H	1.71865500	1.62996100	0.94402300
H	-1.22682500	0.58511300	1.51293800
H	-1.66182800	0.21741600	-1.51978000
H	0.71521800	-0.77757800	1.49480600
O	-0.17479800	1.64245800	0.06528500
O	-3.02910900	-0.19921300	-0.09682100
O	-0.23950100	-1.85735100	0.01008200
H	0.45083100	-2.52562000	0.04993600
O	2.60338900	-0.62903000	-0.08789400
H	3.25292600	-0.38702100	-0.75213400
H	1.62255900	2.13621200	-0.76520200

Product (H_2O)

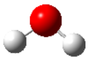

H	1.25440900	-2.44994500	1.61081100
O	2.04838200	-2.91340400	1.33735600
H	2.55921300	-3.03028400	2.14067700

TS-FF1

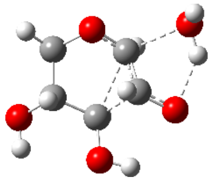

C	-1.61434800	-0.31073800	-0.29949600
C	-1.03417000	-1.68035500	0.05314700
C	0.97770400	-0.66631000	0.24546900
C	0.85382300	0.64933100	-0.33187200
C	-0.70028100	0.76892400	0.25687800
H	-1.11354400	-1.85685700	1.13111800
H	-1.50164800	-2.49400600	-0.49317800
H	-1.66379400	-0.19850700	-1.38940200
H	1.18476800	-0.76797000	1.31274000
H	0.67641000	0.57547400	-1.42420000
H	-0.67570200	0.72026300	1.35390900
O	0.36649000	-1.68087400	-0.34904400
O	3.12627400	-0.58646600	0.13852800
O	1.61685500	1.60899400	0.11804700
H	2.95426200	0.39236900	0.18945300
O	-1.12408200	2.02665300	-0.16356200
H	-0.36857100	2.62059700	-0.01692400
O	-2.90064900	-0.25567900	0.27409600
H	-3.23003700	0.64304800	0.16655700
H	3.50736900	-0.74896000	-0.72944300

Ring contraction (β-D-xylopyranose ⟶ FF2 + H_2O)

Product (FF2)

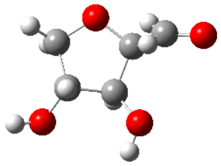

C	-1.51972900	-0.03512900	-0.23308700
C	-1.11301600	-1.48369000	0.01431400
C	2.06258900	-0.29449100	0.45442500
C	0.83314300	-0.26591600	-0.42949500
C	-0.22973600	0.69037100	0.12378500
H	-1.18426900	-1.70412700	1.08623800
H	-1.73003300	0.13916000	-1.29494900
H	1.94927200	-0.93128500	1.35721800
H	1.11262700	0.03869900	-1.44197300
H	-0.17633500	0.73570800	1.22251800
O	0.23773400	-1.55946200	-0.43312300
O	3.06617200	0.31792700	0.23089500
O	-0.08325100	1.96211200	-0.44248200

H	-0.70423700	2.55712900	-0.01234600
O	-2.56082100	0.44834000	0.58599700
H	-3.40931100	0.17167900	0.23252600
H	-1.69749900	-2.21601100	-0.54351400

TS-FF2

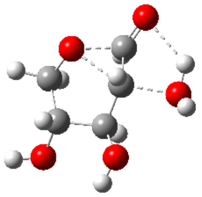

C	1.43570100	-0.86706500	0.34936900
C	0.74943800	0.27158700	-0.30880100
C	-0.56580800	0.78208100	0.17776400
C	-1.62050000	-0.25196000	-0.23104200
C	-1.02522600	-1.63107500	0.05219700
O	2.63323800	-1.04949900	0.05710100
O	1.92770300	1.57823700	0.26490900
O	-0.79971500	2.04782300	-0.39835500
O	-2.79432300	0.08203400	0.48363400
O	0.22841000	-1.66253500	-0.57022900
H	1.05590800	-1.12618500	1.35427500
H	0.98854800	0.39234800	-1.35889300
H	-0.55486200	0.84827900	1.27332900
H	-1.77652800	-0.14805400	-1.31215700
H	-0.96035100	-1.78464900	1.14064900
H	2.71487200	0.99832200	0.16522900
H	1.98713200	2.32548200	-0.34194600
H	-1.68670800	2.32185300	-0.14178500
H	-3.54494900	-0.38190100	0.10494800
H	-1.65323700	-2.42433300	-0.36539100

Elimination (β-D-xylopyranose ⟶ AXP1 + H_2O)

Product (AXP1)

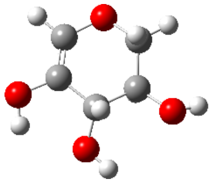

C	1.17875700	-0.16563900	-0.25723200
C	1.24443000	1.26935100	0.25098600
C	-1.05324100	1.42974100	-0.17099100
C	-1.23064700	0.13965300	0.10580800
C	-0.08032700	-0.80883300	0.28815600
H	1.20370200	1.26805700	1.34816300
H	2.16287300	1.76156800	-0.07077900
H	1.11651900	-0.16825200	-1.35204100
H	-1.87898100	2.10789300	-0.33463000

H	0.07089500	-1.02836100	1.35456700
O	0.17178900	2.03610500	-0.26715100
O	-2.48163500	-0.38791800	0.26045500
H	-2.43952900	-1.30441700	-0.03910400
O	-0.41569800	-2.00641600	-0.39613600
H	0.26903400	-2.65610400	-0.21141600
O	2.26971000	-0.94557100	0.19959000
H	3.05544500	-0.73144700	-0.30925300

TS-AXP1

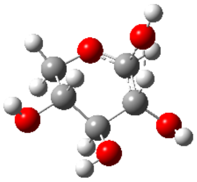

C	1.28593000	-0.22360200	0.23288400
C	0.99053800	-1.49768600	-0.54989500
C	-1.28923200	-0.84573600	-0.44768700
C	-0.99048300	0.53243700	-0.18450000
C	0.45841300	0.92072600	-0.33045300
H	1.38623400	-1.40775400	-1.56396200
H	1.42440400	-2.37351500	-0.06988800
H	1.01781200	-0.35156900	1.28588200
H	-2.31226700	-1.14393300	-0.63919800
H	-1.23183700	0.27096600	1.02915500
H	0.72636000	1.07913800	-1.38625100
O	-0.42033800	-1.78334000	-0.66431200
O	-1.49150100	-0.89646500	1.69353900
H	-1.88704000	-1.28583700	2.48052100
O	-1.92386900	1.40604200	-0.76281900
H	-2.05023200	2.13824300	-0.15407400
O	0.66148500	2.10987500	0.39996500
H	1.59126400	2.34451500	0.31739800
O	2.65559700	0.10758100	0.07235500
H	3.16113400	-0.24358900	0.80834400

Elimination (β-D-xylopyranose ⟶ AXP2 + H_2O)

Product (AXP2)

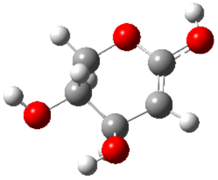

C	-1.07566600	-0.53305700	-0.27914500
C	-0.04602900	-1.53217500	0.22802700
C	1.54375600	0.17377100	0.00987400
C	0.65168100	1.11971100	0.30264400
C	-0.81454100	0.81940900	0.36148000

H	-0.08306400	-1.58211900	1.32195900
H	-0.21008600	-2.52736900	-0.18462900
H	-0.97061600	-0.41486200	-1.36417700
H	0.99362800	2.12997000	0.47294100
H	-1.16697400	0.76594500	1.40233100
O	1.26153500	-1.14158400	-0.18565400
O	2.85895300	0.40728600	-0.14017600
H	3.30976600	-0.44278700	-0.18242600
O	-1.52378700	1.84298900	-0.31443700
H	-2.46167800	1.65251700	-0.21106800
O	-2.39167400	-0.92365300	0.07209700
H	-2.71150500	-1.58116200	-0.54995500

TS-AXP2

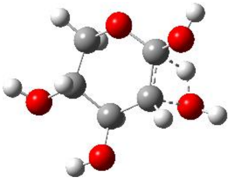

C	-1.36970400	-0.58128500	-0.21315300
C	-0.51714200	-1.61622800	0.51548900
C	1.44120700	-0.40393100	0.00381700
C	0.68289000	0.80474400	-0.22830900
C	-0.79859400	0.79942400	0.06473600
H	-0.47655900	-1.37294700	1.58764000
H	-0.93892400	-2.61624900	0.39839800
H	-1.33194900	-0.77886100	-1.29172200
H	1.71686200	0.59495100	1.23888900
H	0.98957500	1.50718100	-1.00118300
H	-0.95289600	0.99800200	1.13235900
O	0.76517000	-1.64646900	-0.05328800
O	2.57138500	-0.47732300	-0.81309000
H	3.23204800	-1.00082200	-0.35473400
O	1.36870400	1.65958500	1.16490500
H	2.09050200	2.25386400	0.90774300
O	-1.41147600	1.79904300	-0.71608400
H	-2.35964500	1.73454300	-0.55996200
O	-2.70848000	-0.53608800	0.25217400
H	-3.18675600	-1.30459500	-0.06831600

Elimination (β-D-xylopyranose ⟶ AXP3 + H_2O)

Product (AXP3)

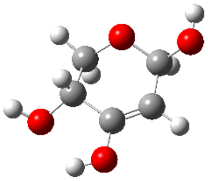

C	-1.10659900	-0.55398000	-0.37992700

C	-0.09260600	-1.52971900	0.20584600
C	1.62278600	0.07653400	0.37067200
C	0.56832600	1.14003400	0.30498000
C	-0.68465300	0.85156400	-0.03187000
H	-0.18988500	-1.52337400	1.29979000
H	-0.25924500	-2.54477700	-0.15795100
H	-1.13674900	-0.65322000	-1.47206300
H	1.89309100	-0.12796700	1.42069400
H	0.86105300	2.15229800	0.55258300
O	1.20058300	-1.15067600	-0.20339100
O	2.74090500	0.51050600	-0.33392900
H	3.40388900	-0.18602200	-0.29250700
O	-1.65307100	1.79307800	-0.12026500
H	-2.50350400	1.34076200	-0.05070000
O	-2.40883800	-0.74071600	0.16410800
H	-2.84801900	-1.46193800	-0.29215200

TS-AXP3

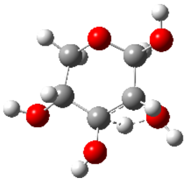

C	-1.28508600	-0.78159200	-0.23305400
C	-0.25543900	-1.69845000	0.42169900
C	1.54844000	-0.24488500	0.16894700
C	0.60699100	0.88222300	-0.23973100
C	-0.79697800	0.63499400	-0.23018100
H	-0.22119400	-1.48990300	1.49982700
H	-0.51403700	-2.74759200	0.27609300
H	-1.44893900	-1.09848600	-1.27280200
H	1.70698300	-0.21701000	1.25565000
H	1.03022500	1.59446600	-0.94323400
H	-0.45388800	1.38210500	1.02698400
O	1.01481900	-1.50236700	-0.17397100
O	2.73393600	-0.06522900	-0.52773700
H	3.29375400	-0.82974100	-0.36010200
O	0.60708700	1.88424600	1.23773700
H	0.62118100	2.83340100	1.04717000
O	-1.52378600	1.49227700	-1.08810900
H	-2.39995000	1.59102300	-0.70691800
O	-2.52432600	-0.82610100	0.47258600
H	-3.02794200	-1.58592500	0.16974700

Elimination (β-D-xylopyranose ⟶ AXP4 + H_2O)

Product (AXP4)

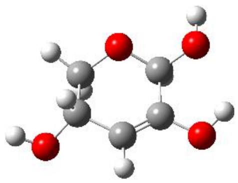

C	-1.60516600	0.06556200	-0.40374100
C	-1.10293800	-1.22263100	0.24691800
C	1.12904000	-0.56284300	0.33457300
C	0.68620100	0.85351600	0.04709800
C	-0.56895000	1.14110200	-0.27871400
H	-1.15511900	-1.09678600	1.33655900
H	-1.70906600	-2.08302600	-0.03909100
H	-1.81404000	-0.11536400	-1.46629300
H	1.22860600	-0.70164200	1.42488100
H	-0.86544700	2.16426500	-0.47661800
O	0.22365400	-1.50810300	-0.16351300
O	2.36592200	-0.73664900	-0.28756900
H	2.68762200	-1.62262200	-0.09656400
O	1.64580100	1.79805300	0.20736600
H	2.50257300	1.38080700	0.05485400
O	-2.79041800	0.50753400	0.24719100
H	-3.53612600	0.00213200	-0.08542700

TS-AXP4

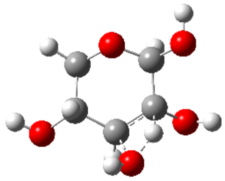

C	1.53050300	-0.22490600	0.23015100
C	0.91748200	-1.56704800	-0.17190600
C	-1.29697100	-0.80094800	-0.16586700
C	-0.80526000	0.62028300	-0.26860500
C	0.60314500	0.86943800	-0.25141200
H	0.84813700	-1.63604600	-1.26608800
H	1.52420000	-2.39762700	0.19476600
H	1.58628100	-0.21611100	1.32432900
H	-1.51061800	-1.18234500	-1.17925100
H	-0.52380300	1.45851800	0.94841100
H	1.03064700	1.53389800	-0.99615900
O	-0.34541300	-1.66524300	0.43939800
O	-2.45469200	-0.82936700	0.60666600
H	-2.83375900	-1.71214300	0.55095400
O	-1.50197900	1.34662300	-1.25981200
H	-2.29757700	1.70394000	-0.86052400
O	0.48569200	2.06675500	1.06365000
H	1.03777500	1.97607500	1.85517000
O	2.79809500	0.00481400	-0.34901800

H	3.42571700	-0.63332600	0.00012400

Elimination (β-D-xylopyranose ⟶ AXP5 + H_2O)

Product (AXP5)

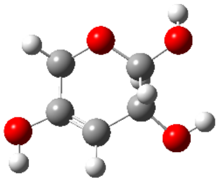

C	-1.55443100	0.14313300	-0.02794700
C	-1.13457100	-1.29412200	0.11818900
C	1.06718000	-0.51929700	0.25887200
C	0.78024800	0.83683400	-0.36642100
C	-0.68826600	1.12493600	-0.26157000
H	-1.28675600	-1.60570900	1.16162800
H	-1.75926800	-1.92206900	-0.51841100
H	0.91353100	-0.45308000	1.34861100
H	1.10229100	0.78965500	-1.41524800
H	-1.00450900	2.15724600	-0.37202700
O	0.20455600	-1.49682200	-0.27729500
O	2.37694600	-0.85926100	-0.05196400
H	2.56840200	-1.72760400	0.31459100
O	1.48682200	1.85583000	0.31692300
H	2.42613500	1.66775700	0.22223500
O	-2.89864000	0.27367500	0.12360800
H	-3.13835000	1.20526800	0.10617100

TS-AXP5

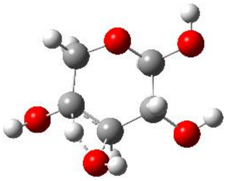

C	1.43052100	-0.41980800	-0.08948500
C	0.62576500	-1.70243600	0.08418800
C	-1.36071500	-0.50292000	-0.17637000
C	-0.73974500	0.86133400	0.10243400
C	0.71182100	0.80118000	-0.30889700
H	0.56715000	-2.20815600	-0.89169400
H	1.09550700	-2.38605600	0.79096800
H	1.81414400	0.53990000	1.02795700
H	-1.29269400	-0.75291900	-1.24794000
H	-0.83515800	0.99768400	1.18694300
H	0.98098400	1.44066600	-1.14410000
O	-0.68413400	-1.46429200	0.58361000
O	-2.68425300	-0.42573600	0.24396500
H	-3.09112800	-1.29262600	0.15254500

O	-1.37237700	1.89872600	-0.60421700
H	-2.31626400	1.83769200	-0.42097000
O	1.62714400	1.69282700	0.93987200
H	1.14661300	2.17083600	1.63299500
O	2.49588900	-0.62972500	-1.00136700
H	3.32277900	-0.63990600	-0.51793400

Elimination (β-D-xylopyranose ⟶ AXP6 + H_2O)

Product (AXP6)

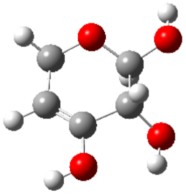

C	0.99900900	1.47450400	0.12806700
C	-0.41415700	1.98011600	0.24004400
C	-1.17599700	-0.24195400	0.21322800
C	0.13852500	-0.83756300	-0.26987900
C	1.23184800	0.19122600	-0.12366000
H	-0.66598100	2.19365000	1.28985000
H	-0.53909400	2.90370700	-0.32672800
H	1.81140500	2.18439000	0.25064700
H	-1.15918200	-0.20245200	1.31504400
H	0.02470700	-1.11331800	-1.32627500
O	-1.34342000	1.06118100	-0.29848800
O	-2.20898300	-1.02948100	-0.26067500
H	-3.04016000	-0.64174600	0.02856500
O	0.41017200	-1.97168800	0.52381500
H	1.31612600	-2.24407500	0.34865600
O	2.45725000	-0.39107900	-0.26919300
H	3.15430400	0.25669900	-0.13098500

TS-AXP6

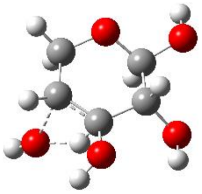

C	-1.24084000	-0.82112400	-0.52519500
C	-0.21327200	-1.88853900	-0.21884200
C	1.40514400	-0.25845300	0.29425900
C	0.58691400	0.91527100	-0.22084300
C	-0.86973100	0.52359900	-0.32008800
H	-0.33036400	-2.21349500	0.82087800
H	-0.36009000	-2.74693000	-0.87360800
H	-2.10402800	-1.07276400	-1.12897200
H	1.16258200	-0.43846000	1.35538500
H	0.96546200	1.16829900	-1.22027300

H	-1.42256900	0.24615300	0.87566700
O	1.09596000	-1.42178500	-0.45484700
O	2.74234400	0.02788700	0.10986800
H	3.26154000	-0.70871300	0.44515200
O	0.76480300	1.99766400	0.66384900
H	0.20326600	2.71413900	0.35259500
O	-1.61892400	1.54353100	-0.93242400
H	-2.49304900	1.56287900	-0.53200400
O	-2.30666400	-0.68893200	1.11005600
H	-2.73112800	-1.21656900	1.80463600

Elimination (β-D-xylopyranose ⟶ AXP7 + H_2O)

Product (AXP7)

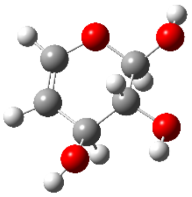

C	-1.03767700	1.49752100	-0.16083000
C	0.18192300	1.97919900	0.06858000
C	1.21027000	-0.12722800	-0.29239500
C	-0.07807000	-0.75188700	0.21216500
C	-1.26736600	0.02381200	-0.33611700
H	0.38880200	3.02972700	0.22902300
H	-1.87536700	2.18200800	-0.20897200
H	1.22333000	-0.12394200	-1.39219100
H	-0.08446300	-0.66974400	1.30717600
H	-1.37960000	-0.23545900	-1.39770600
O	1.31258500	1.23458700	0.14001600
O	2.28100500	-0.81344700	0.23533000
H	3.09269200	-0.43963200	-0.11958600
O	-0.11450500	-2.09206200	-0.20722800
H	-0.96133500	-2.45066600	0.07753100
O	-2.39627200	-0.46242700	0.38207900
H	-3.19939500	-0.12073900	-0.01800500

TS-AXP7

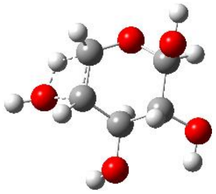

C	-1.18883000	-0.37149800	-0.57716200
C	-0.59112500	-1.59923100	-0.18425700
C	1.46471500	-0.54990300	0.33722100
C	0.89989500	0.78357700	-0.18479700
C	-0.60308500	0.83378300	0.09038000
H	-1.92496200	-1.55412900	0.42566700
H	-0.43326600	-2.37855600	-0.92320400
H	-1.62391900	-0.23485600	-1.56220100
H	2.05776700	-0.35302300	1.23391500
H	1.06849500	0.83582900	-1.26764200
H	-0.74590700	0.74535100	1.17195500
O	0.45260100	-1.44143900	0.75667300
O	2.24218200	-1.10208800	-0.68778700
H	2.65300100	-1.90314100	-0.34862800
O	1.56136800	1.83659100	0.47530700
H	1.13570900	2.65615000	0.20201800
O	-1.09692000	2.06315300	-0.39373800
H	-1.97025700	2.20879500	-0.01888100
O	-2.72886300	-0.63725300	0.38131700
H	-3.52734900	-0.82592600	-0.13189200

2. Optimized Cartesian coordinates for minima and TSs of 2-O-acetyl-β-D-xylopyranose's initial reactions at the M06-2X/6-311++G(d,p) level

Ring-opening (2-O-acetyl-β-D-xylopyranose ⟶ OADXP)

Reactant (2-O-acetyl-β-D-xylopyranose)

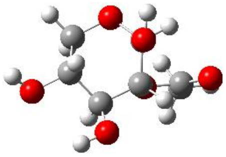

C	0.94247300	-1.88759700	-0.00260700
C	-0.32336700	-1.94751500	-0.85195400
C	-1.13084900	-0.34696500	0.75927600
C	0.12839700	0.45862800	0.44041000
C	1.34579200	-0.43098100	0.15647700
H	-0.11989700	-1.46794000	-1.81695700
H	-0.61872200	-2.98256100	-1.03585800
H	0.76876100	-2.32032000	0.98570900
H	-2.00023300	0.31212500	0.79529000
H	0.34421300	1.12385900	1.27635100
H	1.75796900	-0.11210300	-0.80961500
O	-1.43034100	-1.30257700	-0.22580400
O	-0.90004900	-0.94053900	2.01022700
H	-1.70648100	-1.38976500	2.27900700
O	-0.11128000	1.22167900	-0.75060700
O	2.29428900	-0.25155400	1.18356200
H	3.03530500	-0.83534000	0.99218800
O	2.02930700	-2.52950900	-0.65443800
H	1.95140200	-3.48004600	-0.54387600
C	-0.83562800	2.35336300	-0.61909200
C	-0.98909100	3.04502700	-1.94328500
H	-0.00455500	3.26780300	-2.35597100
H	-1.50043600	2.38047500	-2.64107800
H	-1.55912500	3.95993200	-1.80697000
O	-1.28297100	2.73063100	0.42811900

Product (OADXP)

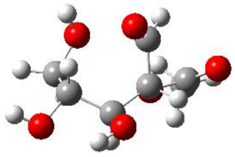

C	0.85112200	-1.82815700	-0.09176100
C	-0.22503100	-1.89193400	-1.17586300
C	-0.88821500	0.12795700	1.38608300
C	0.27610500	0.62275100	0.53398300
C	1.34822500	-0.41336000	0.19151400
H	0.03807700	-1.23326400	-2.00546100
H	-0.27970300	-2.91677600	-1.56257800
H	0.47090700	-2.25741400	0.84249600
H	-1.86116400	0.59333600	1.14827300
H	0.76815800	1.42336500	1.09606800
H	1.83297300	-0.05911700	-0.72924500
O	-1.48798300	-1.45896800	-0.70112100
O	-0.76633200	-0.68001100	2.26365700
H	-1.81517800	-2.09704000	-0.05976900
O	-0.22190000	1.14060200	-0.70155800
O	2.26646300	-0.42111800	1.25914900
H	2.89293900	-1.13155200	1.08417400
O	2.02387600	-2.52075000	-0.51272000

H	1.88087600	-3.46766200	-0.44391900
C	-0.84190300	2.33891800	-0.64985400
C	-1.31769500	2.75412700	-2.01112300
H	-0.46785600	2.81694800	-2.69192900
H	-2.00109000	1.99731500	-2.39885000
H	-1.81692300	3.71651400	-1.94016500
O	-0.97651500	2.95939500	0.36835300

TS-OADXP

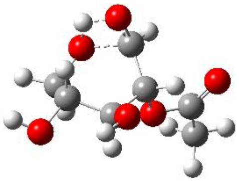

C	0.95037600	-1.90784700	0.08510000
C	-0.34066900	-2.00236100	-0.72661300
C	-1.09286100	-0.23598500	1.08895200
C	0.15343300	0.46562200	0.56972400
C	1.34618600	-0.44847300	0.25993100
H	-0.26480200	-1.40656900	-1.64158000
H	-0.56416200	-3.03827200	-1.00093900
H	0.80137500	-2.34138900	1.08153800
H	-2.00052900	0.36857600	0.94840300
H	0.45232800	1.19548500	1.32873900
H	1.74627100	-0.11661000	-0.71081400
O	-1.44549400	-1.51112300	0.04131700
O	-1.00285100	-1.02061600	2.12679000
H	-1.38581800	-1.85129400	1.15805300
O	-0.18190400	1.13658500	-0.65376000
O	2.30729800	-0.30048000	1.27565600
H	3.02550700	-0.91558100	1.06081400
O	2.03573500	-2.52130000	-0.58921500
H	1.98020500	-3.47836000	-0.46225500
C	-0.81702800	2.32675600	-0.53171300
C	-1.08388900	2.92815900	-1.88482600
H	-0.13990500	3.07562600	-2.41521100
H	-1.69497200	2.24320600	-2.47790300
H	-1.59835000	3.87931700	-1.75980100
O	-1.11146400	2.81133600	0.52939000

Elimination (2-O-acetyl-β-D-xylopyranose ⟶ AXPL1 + H_2O)

Product (AXPL1)

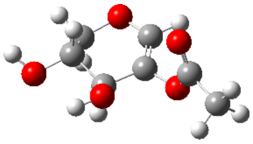

C	0.75829700	-1.74699100	-0.19790100
C	-0.28546700	-2.63380400	-0.86509200
C	-1.58994000	-0.71126700	-1.14096100
C	-0.50583300	0.04945700	-1.24995400
C	0.88427200	-0.45258100	-0.98228400
H	0.00017400	-2.81132000	-1.90923500
H	-0.37753500	-3.59184300	-0.35339200
H	0.43738400	-1.49059400	0.81854500
H	-2.58831500	-0.32395000	-1.29426000
H	1.40503100	-0.66752100	-1.92680800
O	-1.57313500	-2.03190700	-0.82300900
O	-0.60044000	1.37813200	-1.62694400
O	1.59202000	0.53454700	-0.26350200
H	2.47751700	0.19229800	-0.10654500
O	2.03724500	-2.35674800	-0.19946100
H	2.09147900	-2.99659900	0.51437400
C	-0.77014600	2.29604500	-0.62113200
C	-0.50984500	3.67701900	-1.14450300
H	0.56248200	3.77571300	-1.32398900
H	-1.02423800	3.82404600	-2.09389900
H	-0.83171400	4.41004200	-0.40977300
O	-1.08472400	2.00254100	0.49031200

TS-AXPL1

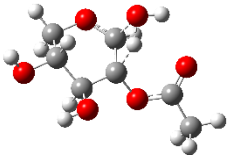

C	1.09569200	-1.98032000	0.08125100
C	-0.18210200	-2.65226500	-0.39400100
C	-1.28307000	-0.58637100	0.06886100
C	-0.03855000	0.16855600	-0.10984000
C	1.18719700	-0.58344700	-0.51391300
H	-0.15182200	-2.74950300	-1.48362600
H	-0.29833800	-3.64302200	0.04503200
H	1.10380000	-1.89207000	1.17400200
H	-2.21014700	-0.10919300	-0.23465500
H	-0.41380100	0.22328100	1.38580300
H	1.26926600	-0.68667000	-1.60934800
O	-1.35879100	-1.92307200	-0.02850900
O	-1.35766600	-0.28235500	1.78435700
H	-1.96124500	0.46508600	1.91497600
O	-0.18329300	1.34593200	-0.91396700
O	2.33311400	0.09069400	-0.02871500
H	3.09894100	-0.40985200	-0.32921200
O	2.22197300	-2.70880400	-0.37847100
H	2.44281900	-3.39675700	0.25308500
C	-0.88943800	2.36015100	-0.41738400
C	-0.73315500	3.60104900	-1.24923600

H	0.27164900	3.99822600	-1.09204300
H	-0.83397600	3.36147300	-2.30734000
H	-1.47206300	4.33821200	-0.94668500
O	-1.57211300	2.27925000	0.57808300

Elimination (2-O-acetyl-β-D-xylopyranose ⟶ AXPL2 + CH_3COOH)

Product (AXPL2)

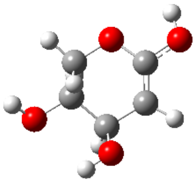

C	1.10759800	-2.19603200	0.14139300
C	-0.16520900	-1.73041300	-0.55050700
C	-0.48140500	-0.28221200	1.26294000
C	0.83029600	-0.12253400	1.43684000
C	1.82363900	-0.99213800	0.72940600
H	0.07843100	-0.98445100	-1.31519300
H	-0.70129200	-2.55914900	-1.01241200
H	0.85221600	-2.86964600	0.96799600
H	1.18122800	0.63250800	2.12481500
H	2.30154300	-0.45292300	-0.10202800
O	-1.06061600	-1.15993000	0.40148500
O	-1.41482400	0.41180200	1.93666400
H	-2.26851700	0.24074800	1.52483400
O	2.82085400	-1.39866400	1.65025400
H	3.47370100	-1.90385500	1.15515100
O	1.99574300	-2.81506200	-0.77285300
H	1.73299800	-3.72771000	-0.91271200

Product (CH_3COOH)

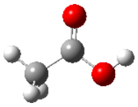

H	0.14301300	1.39629600	-0.09265200
O	0.02070100	1.95991900	-0.86833000
C	-1.30825000	2.10164900	-1.05620500
C	-1.60655100	2.96667300	-2.24575800
H	-1.15361700	3.94870100	-2.10361500
H	-1.16229800	2.52310900	-3.13775700
H	-2.68229900	3.05924400	-2.36453400
O	-2.11655400	1.58337100	-0.33820000

TS-AXPL2

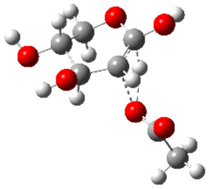

C	1.21185000	-2.08818300	0.08947800
C	-0.14808400	-2.09704700	-0.59974800
C	-0.89914800	-0.47587600	0.92585800
C	0.39672700	0.05227700	1.05864100
C	1.58045900	-0.65371400	0.44109400
H	-0.14011800	-1.42385000	-1.46656500
H	-0.42583100	-3.09827200	-0.92595600
H	1.14963700	-2.67130800	1.01669900
H	-0.74611200	0.39368600	0.06430700
H	0.56742600	0.92472900	1.68780900
H	1.84616100	-0.12554700	-0.47897500
O	-1.15023200	-1.69590600	0.33163200
O	-1.85449600	-0.12919100	1.82515500
H	-2.71963200	-0.24620400	1.41725400
O	0.26513600	1.58995200	-0.53105600
O	2.61014900	-0.59160200	1.39752600
H	3.42986900	-0.84560100	0.96329400
O	2.24103300	-2.56133400	-0.75327200
H	2.23878100	-3.52153500	-0.76670300
C	0.26177500	2.78266500	-0.02022700
C	-0.11546700	3.89565900	-0.98189300
H	0.63046000	3.94519500	-1.77727100
H	-1.07576600	3.67078600	-1.44856500
H	-0.16226600	4.84726900	-0.45689600
O	0.54161900	3.02191600	1.14918200

Elimination (2-O-acetyl-β-D-xylopyranose ⟶ AXPL3 + CH_3COOH)

Product (AXPL3)

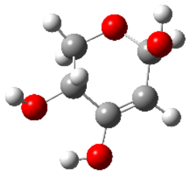

C	0.78260500	-1.92642600	0.34590600
C	-0.25307600	-1.38199500	-0.63938500
C	-1.05910400	-0.14164300	1.28792500
C	0.38226500	0.12641500	1.59748300
C	1.27468600	-0.76591100	1.17869000
H	0.28149100	-0.73861400	-1.34651500
H	-0.72067500	-2.19986300	-1.19259600
H	0.33772400	-2.67862100	1.00604200

H	-1.65146400	0.77529000	1.33315300
H	0.65472600	0.97231100	2.21249600
O	-1.29100600	-0.64991700	-0.01441500
O	-1.54244100	-1.07129400	2.23826200
H	-2.46783700	-1.23841600	2.03016700
O	2.59214200	-0.70705400	1.44438500
H	3.02983200	-1.37180300	0.89545600
O	1.89608600	-2.46181700	-0.35563000
H	1.74755100	-3.39000000	-0.54582200

TS-AXPL3

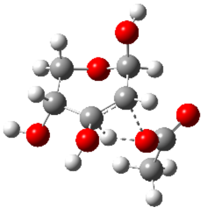

C	0.99079800	-1.84021500	-0.12398000
C	-0.48159900	-2.25370600	-0.17640200
C	-1.30720800	-0.38304200	0.97680900
C	0.09996700	0.10103700	1.23863500
C	1.17290600	-0.51782100	0.58867200
H	-0.68116900	-2.81364900	-1.09013400
H	-0.71055300	-2.88332000	0.68824400
H	1.54802600	-2.59251200	0.44713600
H	-1.96908000	0.46835600	0.82104600
H	0.26389700	0.80771100	2.04139500
H	0.84159100	0.40062000	-0.23746200
O	-1.36260900	-1.14065200	-0.19723300
O	-1.64314100	-1.12666200	2.12088600
H	-2.60156400	-1.17079100	2.18515600
O	0.07752000	1.60436000	-0.18910100
O	2.44704300	-0.26834500	1.08434200
H	3.05346300	-0.23693300	0.33748800
O	1.59376800	-1.66820300	-1.40155300
H	1.71658600	-2.52617700	-1.81632300
C	-0.91569700	2.14407100	-0.86170200
C	-0.59997700	2.41988300	-2.31545200
H	0.30502500	3.02377400	-2.39112800
H	-0.41045600	1.46995800	-2.82028500
H	-1.43823600	2.92982900	-2.78458500
O	-1.99843000	2.38126400	-0.36345600

Elimination (2-O-acetyl-β-D-xylopyranose ⟶ AXPL4 + H_2O)

Product (AXPL4)

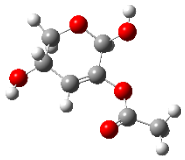

C	1.02299600	-2.02012800	-0.32324400
C	-0.32501200	-2.71932700	-0.27098400
C	-1.56624200	-0.73239900	-0.02481800
C	-0.35723900	0.00758300	-0.55564600
C	0.83461700	-0.56649100	-0.67950200
H	-0.70621600	-2.82008300	-1.29560000
H	-0.24221400	-3.70865300	0.17568100
H	1.51537000	-2.10517600	0.65359700
H	-2.27629600	-0.90367700	-0.85190500
H	1.68879900	-0.01821900	-1.05025800
O	-1.21377400	-1.97143600	0.53555900
O	-2.14736000	0.04172600	0.97083300
H	-2.85412500	-0.47912200	1.36502600
O	-0.73971500	1.27846900	-0.92158800
O	1.77787700	-2.70104000	-1.31269000
H	2.66674600	-2.33819100	-1.33734500
C	0.13172700	2.29474600	-1.15562900
C	-0.63233300	3.54996700	-1.46290800
H	-1.27796500	3.38339900	-2.32602500
H	-1.27012500	3.80091700	-0.61444500
H	0.06870700	4.35544000	-1.66346100
O	1.32170100	2.18689900	-1.11382800

TS-AXPL4

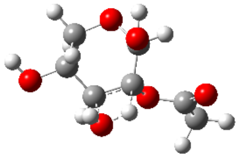

C	0.88762700	-2.00425500	-0.12093200
C	-0.44598900	-2.29490200	-0.83134500
C	-1.22065600	-0.44535900	0.51808900
C	-0.03203200	0.31075600	0.00292400
C	1.09598100	-0.52838100	-0.32089700
H	-0.34549400	-1.99589800	-1.88275600
H	-0.65824100	-3.36659300	-0.79732000
H	0.78326500	-2.24498900	0.94092800
H	-2.12381100	0.16362300	0.47709400
H	1.18348200	0.60995600	1.04382100
H	1.65613000	-0.29303500	-1.22195600
O	-1.53931000	-1.62627900	-0.23264100
O	-0.93385500	-0.83390400	1.84856200
H	-1.66918500	-1.37663800	2.15028000
O	-0.33508000	1.22607600	-1.04999400
O	2.13750800	0.06691100	0.86286500

H	2.34704400	-0.57569400	1.55933000
O	1.96670500	-2.69858100	-0.71077900
H	1.85896100	-3.64096800	-0.55604300
C	-0.92821600	2.38477900	-0.70561300
C	-1.08701300	3.27488400	-1.90911100
H	-0.10570100	3.49806700	-2.33024900
H	-1.66697200	2.75806200	-2.67468200
H	-1.58733100	4.19320300	-1.61365000
O	-1.28917200	2.64843500	0.40763000

Elimination (2-O-acetyl-β-D-xylopyranose ⟶ AXPL5 + H_2O)

Product (AXPL5)

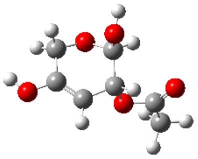

C	1.04348500	-1.43394200	-0.83697600
C	-0.24201600	-2.11746800	-0.46717100
C	-0.99039500	-0.07574500	0.44295800
C	0.11173500	0.74375300	-0.22873000
C	1.21398800	-0.12108800	-0.74235600
H	-0.53809600	-2.80588900	-1.26483300
H	-0.08248100	-2.69877200	0.45070500
H	-1.91265500	0.50495400	0.48889200
H	0.47299100	1.47264400	0.49826000
H	2.15649300	0.33080100	-1.02463000
O	-1.31732300	-1.21874300	-0.31072900
O	-0.52655800	-0.41249700	1.71964700
H	-1.26599200	-0.74199200	2.23771800
O	-0.44288200	1.46180800	-1.35303000
O	2.06337500	-2.25102700	-1.21570700
H	1.73051100	-3.12797100	-1.42502000
C	-1.15120800	2.57136000	-1.07549800
C	-1.62204400	3.23784800	-2.33755800
H	-0.76017100	3.51038400	-2.94785900
H	-2.22752100	2.53729300	-2.91389800
H	-2.20136200	4.12167300	-2.08444900
O	-1.36684400	2.95808900	0.04217600

TS-AXPL5

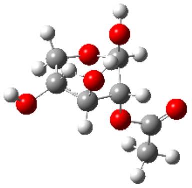

C	0.97726500	-1.78636500	-0.55941800
C	-0.48008200	-2.19506900	-0.72707500
C	-1.09331000	-0.35709000	0.69432000
C	0.15703200	0.44946900	0.34430000
C	1.28189300	-0.47791300	-0.06157300
H	-0.66058500	-2.37103600	-1.78915100
H	-0.70690600	-3.11871500	-0.18436900
H	1.62226800	-1.90997800	0.81953000
H	-1.94337700	0.31164900	0.83934600
H	0.42300800	1.10634400	1.17319200
H	2.14592500	0.02096200	-0.48836400
O	-1.45354600	-1.21234800	-0.33206800
O	-0.77556700	-1.03645000	1.89588400
H	-1.55765100	-1.50318600	2.20475000
O	-0.08912400	1.22902600	-0.82564600
O	1.98368600	-0.99646800	1.45370100
H	1.32564800	-0.88924100	2.16096400
O	1.77676400	-2.20718400	-1.65154900
H	2.27292700	-2.98351700	-1.39001800
C	-0.80155700	2.36586300	-0.65931300
C	-0.98764100	3.07311100	-1.96921900
H	-0.01560000	3.26692000	-2.42356800
H	-1.54772700	2.42723700	-2.64694700
H	-1.52441500	4.00302700	-1.80311500
O	-1.21548100	2.72686200	0.40732200

Elimination (2-O-acetyl-β-D-xylopyranose ⟶ AXPL6 + H_2O)

Product (AXPL6)

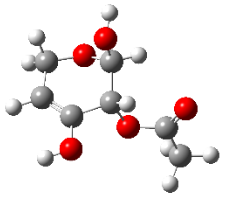

C	0.95741500	-1.92260800	0.93618300
C	-0.31840300	-2.54602400	0.44823400
C	-1.36706600	-0.43631300	0.64022800
C	-0.04789700	0.32539000	0.77587600
C	1.08200600	-0.60768900	1.07354800
H	-0.11346900	-3.26551400	-0.34607000
H	-0.81092700	-3.08003800	1.27023200
H	1.77211400	-2.59199800	1.19610100
H	-2.08657300	0.17440400	0.09145300
H	-0.14876000	1.06667500	1.57062600
O	-1.21578300	-1.60341500	-0.11728700
O	-1.80482700	-0.70422600	1.94382300
H	-2.69980100	-1.05231300	1.89856300

O	0.27202300	0.98826500	-0.45465500
O	2.19274700	0.05964800	1.48791200
H	2.91482500	-0.56175700	1.62052700
C	-0.35523300	2.15824200	-0.68787300
C	0.11943800	2.77125000	-1.97406300
H	1.18779500	2.97880100	-1.90046200
H	-0.02609600	2.06395900	-2.79118700
H	-0.43146500	3.68900400	-2.16092800
O	-1.17902900	2.62262300	0.05207200

TS-AXPL6

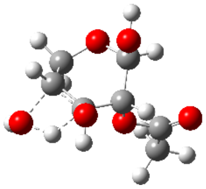

C	0.55911000	-1.52487500	-0.19082200
C	-0.82810900	-1.56519500	-0.80114300
C	-1.46936800	-0.14330900	1.05231700
C	-0.24021800	0.66136900	0.61950500
C	0.93023700	-0.26075200	0.36846800
H	-0.84984700	-0.90581100	-1.67459300
H	-1.10003700	-2.57336000	-1.11082600
H	0.98209600	-2.43691500	0.21615800
H	-2.34948400	0.49584300	1.15828200
H	0.00424000	1.36918000	1.41016200
H	1.56552900	-0.36500300	-1.00331700
O	-1.83019400	-1.13309000	0.11129800
O	-1.11658800	-0.73281300	2.26858800
H	-1.86148400	-1.25993600	2.57066000
O	-0.56757200	1.37355700	-0.59479400
O	1.85485400	-0.33000700	1.43125000
H	2.61828600	0.20464700	1.20770800
O	1.64342100	-1.37493700	-1.59659100
H	1.24571300	-1.40376300	-2.48031500
C	-1.28528100	2.50684400	-0.46299900
C	-1.55215300	3.13254500	-1.80367500
H	-0.61096800	3.29384400	-2.32994300
H	-2.15914300	2.45279100	-2.40393400
H	-2.07613400	4.07411700	-1.66341000
O	-1.65131400	2.94008900	0.59485100

Elimination (2-O-acetyl-β-D-xylopyranose ⟶ AXPL7 + H_2O)

Product (AXPL7)

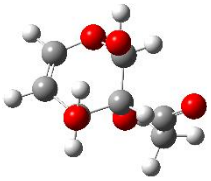

C	0.51112600	-1.52655200	-1.31172000
C	-0.70438200	-1.87100700	-0.89882800
C	-0.66792200	-0.34537800	0.88978200
C	0.26316400	0.53072500	0.05425500
C	1.23827000	-0.33787200	-0.74659400
H	-1.26911600	-2.69724700	-1.31092200
H	0.99006900	-2.11423200	-2.08355700
H	-1.41091000	0.24683000	1.42357000
H	0.80744900	1.20766600	0.71370600
H	1.62039600	0.28042000	-1.56188600
O	-1.41106200	-1.23276200	0.08244100
O	0.16488600	-1.05003000	1.77269900
H	-0.38190700	-1.59571200	2.34551900
O	-0.50626200	1.26815600	-0.89640500
O	2.36764300	-0.69573300	0.02454800
H	2.04957200	-1.22500800	0.76514900
C	-1.19419600	2.33356900	-0.43465600
C	-1.92796700	3.02034900	-1.54959400
H	-1.21503000	3.34865200	-2.30685700
H	-2.61216900	2.31411200	-2.02155100
H	-2.47501900	3.87016600	-1.15081100
O	-1.19672200	2.66217400	0.71968700

TS-AXPL7

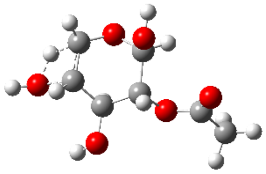

C	0.96769100	-1.81172400	0.48609000
C	-0.26633000	-2.48700700	0.30070500
C	-1.43037300	-0.44836500	0.27760300
C	-0.12653300	0.38319100	0.28876100
C	1.05494100	-0.46527400	-0.17273800
H	0.69819300	-3.12957300	-0.58387600
H	-0.64408100	-3.11931500	1.09799700
H	1.61093000	-1.99312500	1.34082700
H	-2.18633000	0.06081700	-0.32522600
H	0.03778900	0.78259500	1.29108500
H	0.91778800	-0.61880700	-1.24833900

O	-1.24865900	-1.70321300	-0.34960700
O	-1.83156900	-0.59291200	1.60640000
H	-2.68085300	-1.04448300	1.61766400
O	-0.19271200	1.45775900	-0.65092800
O	2.25414000	0.21292300	0.10208000
H	2.94781600	-0.18998600	-0.42825900
O	1.87791000	-2.84031400	-0.75058600
H	2.50501700	-3.48708000	-0.39757700
C	-0.93946700	2.52423100	-0.29621100
C	-0.85101000	3.60729100	-1.33305500
H	0.18212200	3.94966000	-1.40705700
H	-1.13981000	3.20985800	-2.30662900
H	-1.50117600	4.43049100	-1.04972200
O	-1.57636000	2.56701500	0.71890900

3. Optimized Cartesian coordinates for minima and TSs of 4-methoxy-5-carboxy-β-D-xylopyranose's initial reactions at the M06-2X/6-311++G(d,p) level

Ring-opening (4-methoxy-5-carboxy-β-D-xylopyranose ⟶ DGLA)

Reactant (4-methoxy-5-carboxy-β-D-xylopyranose)

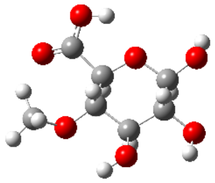

C	-1.35669900	-0.79061400	-0.41907000
C	-0.52005600	-1.91939600	0.21856000
C	1.46303600	-0.55005400	0.26094400
C	0.72188800	0.64017600	-0.32410000
C	-0.75283200	0.53468500	0.02533500
H	-0.64335500	-1.88548800	1.30779900
H	-1.29497000	-0.86683200	-1.51409800
H	1.43186900	-0.50684500	1.35986300
H	0.84435400	0.60913000	-1.41555300
H	-0.85840400	0.60251500	1.11881000
O	0.83561300	-1.76258400	-0.16280000
O	2.75139200	-0.55714400	-0.22206000
H	3.27902200	-1.18140200	0.28371100
O	1.27689800	1.80939800	0.22137200
H	0.71652800	2.54413800	-0.05072100
O	-1.40032000	1.62661200	-0.58913600
H	-2.32942000	1.59033600	-0.33816300
O	-2.69298200	-0.78377500	0.01097100
C	-3.63365600	-1.33347800	-0.90221500
H	-3.44960600	-2.39423600	-1.07740000
H	-4.61290300	-1.21541800	-0.44201400
H	-3.60852500	-0.78477700	-1.85020800
C	-0.96615300	-3.30023100	-0.25497000
O	-1.96509100	-3.82076100	0.14454200
O	-0.18309400	-3.86144700	-1.17910400
H	0.60202300	-3.30489100	-1.30034000

Product (DGL)

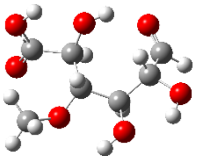

C	-1.25606700	-0.66089800	-0.50379500
C	-0.56177000	-1.99720800	-0.15040000
C	1.87746100	0.13028300	0.63224200
C	0.91504500	0.78968500	-0.34453900
C	-0.55541300	0.56363300	0.09954800
H	-0.19046000	-1.97031400	0.87816700
H	-1.23536400	-0.56447300	-1.59862900
H	2.22415500	0.81270800	1.42854500
H	1.06552900	0.37733000	-1.34933100
H	-0.61187800	0.48195400	1.19537300
O	0.48364500	-2.20988100	-1.08035300
O	2.25483600	-1.01257400	0.58121700
H	1.33222600	-2.03331400	-0.64499700
O	1.25000500	2.15196900	-0.29453400
H	0.45286700	2.65810900	-0.49462100
O	-1.25233300	1.71757500	-0.32393000
H	-2.17918200	1.58244900	-0.09516600

O	-2.57782600	-0.56526300	-0.02760000
C	-3.57500500	-0.97817800	-0.95011200
H	-3.41488800	-2.00120300	-1.29832300
H	-4.52486000	-0.93756800	-0.42107000
H	-3.59788600	-0.30073200	-1.81146700
C	-1.52714500	-3.18503600	-0.21937200
O	-2.34577400	-3.40871400	0.62026300
O	-1.39526700	-3.93559000	-1.31906700
H	-0.59537700	-3.64306200	-1.78505700

TS- DGL

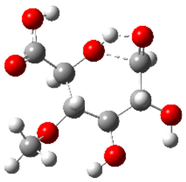

C	-1.26227900	-0.87138500	-0.37477000
C	-0.48280100	-2.02249500	0.25725100
C	1.74979600	-0.15859800	0.00155400
C	0.65094300	0.82106100	-0.38446500
C	-0.71539200	0.44908800	0.17419700
H	-0.84162300	-2.12185400	1.29013200
H	-1.17492900	-0.88950700	-1.46857200
H	2.17703400	0.01482100	0.99508400
H	0.59683100	0.84695500	-1.48014100
H	-0.64495800	0.37693000	1.27020100
O	0.91049500	-1.76120800	0.28949800
O	2.44545900	-0.74623900	-0.90653300
H	1.69133400	-1.68931300	-0.64616900
O	1.06922500	2.06195700	0.13435300
H	0.33140300	2.67460600	0.03258900
O	-1.56298800	1.51890500	-0.18481100
H	-2.44579000	1.30370500	0.13648800
O	-2.61277200	-0.95797800	0.02139300
C	-3.55124500	-1.10900800	-1.03882000
H	-3.37364200	-2.03952800	-1.57878300
H	-4.53537800	-1.13447700	-0.57388200
H	-3.49535300	-0.25735100	-1.72542000
C	-0.72285300	-3.36983300	-0.42523500
O	-1.66070000	-3.59998800	-1.12728800
O	0.20277700	-4.29766200	-0.15231900
H	0.91196300	-3.90398800	0.37442300

Ring contraction (4-methoxy-5-carboxy-β-D-xylopyranose ⟶ FFL1 + H_2O)

Product (FFL1)

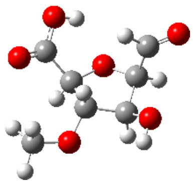

C	-1.79862100	0.03602500	-0.04819700
C	-1.43891300	-1.45074900	0.15659200
C	0.53096500	-0.19642800	0.48297000
C	1.22846200	0.02153400	-0.85417800
C	-0.65061400	0.75463800	0.66133800
H	-2.03736600	-1.86248800	0.97592200
H	-1.79547600	0.29093300	-1.11755500
H	1.26948700	-0.12538300	1.28042400
H	0.63580800	-0.26144100	-1.75070400
H	-0.91088000	0.81546700	1.72465300
O	-0.05517100	-1.50029900	0.51772300
O	2.34484100	0.43748500	-0.94954100
O	-0.34439900	2.01391700	0.12829700
H	-1.08432200	2.60516100	0.29594400
O	-2.99264200	0.44997300	0.54675600
C	-4.17011900	0.04645500	-0.13765300
H	-4.13520700	0.35871100	-1.18627600
H	-4.29686500	-1.03825700	-0.10653000
H	-5.00126000	0.53466600	0.36724000
C	-1.68616100	-2.31401800	-1.08111300
O	-2.73229200	-2.30088000	-1.66095300
O	-0.66031900	-3.07643100	-1.45787100
H	0.07693800	-2.91829900	-0.84750700

TS-FFL1

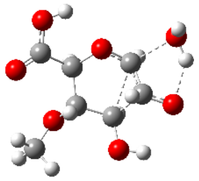

C	-1.84006800	-0.27805000	-0.22561000
C	-1.22007500	-1.66446400	-0.00080800
C	0.80874600	-0.62677700	0.20605400
C	0.65653300	0.66493500	-0.39638400
C	-0.88454700	0.80828300	0.25354300
H	-1.32289200	-1.92528700	1.06071100
H	-2.04683900	-0.13320500	-1.29254300
H	1.03776500	-0.71269300	1.26877600
H	0.42927800	0.57071100	-1.47779900
H	-0.81135000	0.79038300	1.34804000
O	0.19240000	-1.66883300	-0.36316800
O	2.91730200	-0.58119100	0.01582000

O	1.43968600	1.62840700	0.00494100
H	2.76341800	0.40318900	0.06232900
O	-1.29624300	2.05629000	-0.19305500
H	-0.53521200	2.64686000	-0.06293900
O	-3.02148700	-0.26462700	0.53087600
H	3.26373200	-0.75511500	-0.86512200
C	-4.13264100	0.31569800	-0.13840700
H	-3.91468300	1.34778400	-0.42516200
H	-4.39246700	-0.28058700	-1.01793000
H	-4.95959300	0.29892800	0.56865900
C	-1.88484000	-2.75250500	-0.84412800
O	-3.01944900	-2.67456800	-1.19675500
O	-1.11104200	-3.80381800	-1.13141800
H	-0.20050000	-3.63988200	-0.85364300

Ring contraction (4-methoxy-5-carboxy-β-D-xylopyranose ⟶ FFL2 + H_2O)

Product (FFL2)

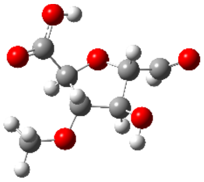

C	1.86466500	-0.26952000	0.57079500
C	0.62079200	-0.53517400	-0.24999900
C	-0.51644600	0.41609500	0.11037700
C	-1.75585600	-0.38441100	-0.28794500
C	-1.30175400	-1.84489400	-0.06276900
O	2.83124900	0.29031700	0.14530700
O	-0.36467700	1.63392800	-0.55786800
O	-2.82442000	0.03022000	0.51068200
O	0.11659800	-1.81695600	0.11937000
H	1.79333700	-0.61992300	1.62032300
H	0.84993000	-0.46933500	-1.31999200
H	-0.55117600	0.54943100	1.20136400
H	-1.97655900	-0.20729800	-1.34943300
H	-1.76484900	-2.24467200	0.84377000
H	-1.03760400	2.24357900	-0.24126300
C	-4.08808900	-0.46393500	0.09223500
H	-4.13690600	-1.55305300	0.17441600
H	-4.83018100	-0.00720000	0.74385100
H	-4.28580300	-0.18879000	-0.94878900
C	-1.67152300	-2.76813700	-1.22456400
O	-0.66209800	-3.47033100	-1.73711500
O	-2.79303500	-2.84437800	-1.63301100
H	0.14086500	-3.25245200	-1.23839000

TS-FFL2

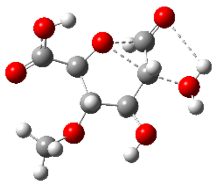

C	1.58976900	-0.91952700	0.25611700
C	0.73802300	0.17698300	-0.29803900
C	-0.59382800	0.50362500	0.30403500
C	-1.65809500	-0.48273600	-0.18727100
C	-1.02960200	-1.88370500	-0.02369000
O	2.72714900	-1.03642800	-0.17477400
O	1.64701400	1.54631700	0.21153500
O	-0.90217400	1.83543400	-0.06445300
O	-2.79752100	-0.15627300	0.56186500
O	0.20023500	-1.84038200	-0.66745300
H	1.28094100	-1.32728300	1.23184800
H	0.83918500	0.30565000	-1.37032200
H	-0.53594100	0.41777500	1.39711600
H	-1.83133700	-0.30078300	-1.25861900
H	-0.95765700	-2.11208400	1.05338500
H	2.54380800	1.40398800	-0.13587900
H	1.23465500	2.35545700	-0.13148000
H	-1.80500100	2.00696400	0.22895700
C	-4.05604300	-0.38242500	-0.06555000
H	-4.28129800	-1.44590600	-0.12126600
H	-4.79745200	0.12462700	0.55008900
H	-4.06260500	0.05407600	-1.07100100
C	-1.88133800	-2.99893600	-0.64998200
O	-1.26435100	-3.63103900	-1.63938400
O	-2.99128600	-3.27159900	-0.28415300
H	-0.37714000	-3.21826200	-1.69926800

<u>Elimination (4-methoxy-5-carboxy-β-D-xylopyranose ⟶ AXPL1 + H_2O)</u>

Product (AXPL1)

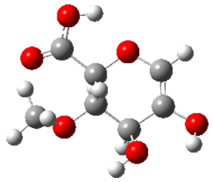

C	-1.40860000	-0.76808900	-0.36304500
C	-0.57456400	-1.90082400	0.26880300

C	1.33209600	-0.52428800	-0.07305000
C	0.61424000	0.57943100	0.10663200
C	-0.88417900	0.54549900	0.19429100
H	-0.63746100	-1.83401700	1.36030100
H	-1.24953300	-0.77165600	-1.45093300
H	2.40913100	-0.52963500	-0.15361500
H	-1.20523700	0.62872900	1.24192200
O	0.77734800	-1.78569500	-0.15453800
O	1.20769800	1.79480100	0.25377000
H	0.58187300	2.45824200	-0.06336600
O	-1.36973700	1.65465400	-0.53994300
H	-2.31878000	1.71334700	-0.39150300
O	-2.77236900	-0.82881800	-0.04869200
C	-3.59911300	-1.40630300	-1.04927300
H	-3.33158200	-2.44598800	-1.24617400
H	-4.61649800	-1.37388000	-0.66461700
H	-3.53866400	-0.82362900	-1.97515500
C	-1.06801700	-3.28247100	-0.14634100
O	-2.02854800	-3.79142900	0.34791600
O	-0.37784800	-3.85698300	-1.13646400
H	0.40900100	-3.32097200	-1.31785300

TS-AXPL1

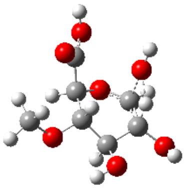

C	1.44275700	-0.13524700	0.31369900
C	1.14215300	-1.51059400	-0.32400600
C	-1.07857400	-0.82409400	-0.70817400
C	-0.90438800	0.46219300	-0.11785400
C	0.51885600	0.95016300	-0.22357800
H	1.91698500	-1.72876600	-1.06428100
H	1.28946100	-0.21102800	1.39538200
H	-2.04328200	-1.14026700	-1.08969200
H	-1.09442200	-0.07961000	1.00545200
H	0.78048600	1.16118900	-1.27114800
O	-0.09342100	-1.60080800	-1.05175200
O	-1.18332000	-1.40768700	1.38389000
H	-1.52637500	-1.97712300	2.08122000
O	-1.89659000	1.36990200	-0.50778300
H	-1.96724700	2.03225400	0.18487000
O	0.63632900	2.12197300	0.54906900
H	1.55225900	2.41318100	0.49331300
O	2.75219000	0.29925000	0.02795400
C	3.76906700	-0.31862700	0.80018800
H	4.69228500	0.21380800	0.57801400
H	3.54249600	-0.25135200	1.86803100
H	3.88622200	-1.37504100	0.54073600
C	1.17190500	-2.63961800	0.70565500

O	0.47472400	-3.70781100	0.32006900
O	1.85779200	-2.59887400	1.68769500
H	0.56544000	-4.37342400	1.01507900

Elimination (4-methoxy-5-carboxy-β-D-xylopyranose ⟶ AXPL2 + H_2O)

Product (AXPL2)

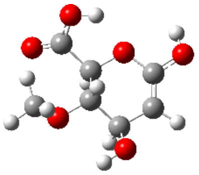

C	-1.39226300	-0.86741400	-0.36483900
C	-0.55668200	-1.98827100	0.28150200
C	1.33318200	-0.62444700	-0.14069800
C	0.63672200	0.49238400	0.04151600
C	-0.85741500	0.47108600	0.12877700
H	-0.58772800	-1.89891000	1.37069800
H	-1.25308800	-0.90636400	-1.45509100
H	1.16228200	1.43374100	0.10854000
H	-1.19103100	0.59357800	1.16924600
O	0.79176000	-1.88294300	-0.18288900
O	2.66108900	-0.67191900	-0.32719800
H	2.97598600	-1.55375600	-0.10008800
O	-1.38117300	1.52377500	-0.65678300
H	-2.33128700	1.54620000	-0.50206900
O	-2.74909000	-0.93822900	-0.02441800
C	-3.58307500	-1.54747400	-0.99876600
H	-3.29993500	-2.58591500	-1.18618700
H	-4.59385000	-1.52904800	-0.59616800
H	-3.55066500	-0.98060700	-1.93557400
C	-1.06440600	-3.37178200	-0.09961700
O	-1.94284300	-3.91511200	0.49705300
O	-0.50151600	-3.90622100	-1.19201000
H	0.24558000	-3.35706600	-1.46820700

TS-AXPL2

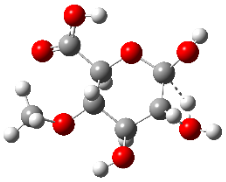

C	-1.46701900	-1.02945600	-0.69393100
C	-0.65579900	-2.17288200	-0.04881200
C	1.33227500	-0.80944000	-0.09100300
C	0.53583400	0.39182900	-0.27397800
C	-0.95662800	0.28246800	-0.10148600

H	-0.77606200	-2.13778200	1.04107100
H	-1.29021200	-1.03874200	-1.77937300
H	1.46224200	0.19110400	1.32372400
H	0.85008000	1.15351000	-0.98824400
H	-1.19223300	0.25228300	0.96960600
O	0.68818200	-2.04107300	-0.43668900
O	2.51789800	-0.74766300	-0.82910900
H	3.19766900	-1.21488200	-0.33998600
O	1.05202700	1.16343400	1.17603000
H	1.75724300	1.80916200	1.00954700
O	-1.56204600	1.39725600	-0.70683200
H	-2.51332500	1.28847800	-0.59803200
O	-2.84000800	-1.06033500	-0.40608100
C	-3.66103600	-1.62103300	-1.42121600
H	-3.41609500	-2.66803900	-1.60534200
H	-4.68510500	-1.55811400	-1.05809000
H	-3.56589300	-1.04465500	-2.34828300
C	-1.13048000	-3.54323600	-0.52564800
O	-2.12300300	-4.06323400	-0.10793800
O	-0.37457000	-4.09483100	-1.47797500
H	0.41296700	-3.53822400	-1.59651500

Elimination (4-methoxy-5-carboxy-β-D-xylopyranose ⟶ AXPL3 + H_2O)

Product (AXPL3)

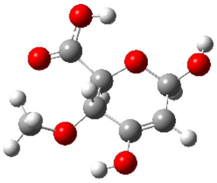

C	-1.33352400	-0.81160200	-0.56611100
C	-0.53217800	-1.89876400	0.17093500
C	1.46474200	-0.57777200	0.29713200
C	0.61678100	0.62827400	0.06067800
C	-0.65062900	0.52185300	-0.32514800
H	-0.69695500	-1.78459200	1.24868200
H	-1.33203200	-1.02041300	-1.64592200
H	1.65180600	-0.72043100	1.37279700
H	1.06549900	1.59725100	0.23647900
O	0.83124500	-1.76871200	-0.17496900
O	2.66297100	-0.44091000	-0.38686000
H	3.26420800	-1.13584700	-0.10177700
O	-1.42724500	1.60179900	-0.55422300
H	-2.34411800	1.31697800	-0.43612000
O	-2.65475700	-0.68213700	-0.09722600
C	-3.66275800	-1.29035200	-0.90007500
H	-3.56722400	-2.37561700	-0.89898100
H	-4.61522300	-1.01148900	-0.45321800
H	-3.61559400	-0.90537600	-1.92460000
C	-0.97148400	-3.30736500	-0.22653600

O	-2.02056900	-3.76812900	0.11599000
O	-0.11837600	-3.96821700	-1.00928600
H	0.67950600	-3.42777600	-1.12115300

TS-AXPL3

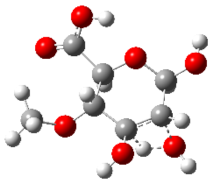

C	-1.37313100	-1.12735900	-0.94485400
C	-0.51856800	-2.25718900	-0.33595300
C	1.44253600	-0.95037700	-0.12956300
C	0.67171900	0.31233200	-0.47992700
C	-0.73672500	0.20591100	-0.65769500
H	-0.62550200	-2.24189600	0.75473700
H	-1.42699200	-1.26801800	-2.03657800
H	1.44081900	-1.10103600	0.95688700
H	1.24420800	1.06276000	-1.01932400
H	-0.48654300	0.72795300	0.73219200
O	0.83575300	-2.08206100	-0.73004100
O	2.72204200	-0.82012600	-0.63393700
H	3.24241000	-1.58199900	-0.36140100
O	0.58196400	1.09026400	1.11681100
H	0.69754500	2.05132700	1.08196400
O	-1.28782100	1.24921800	-1.43324800
H	-2.18008500	1.39742900	-1.10830100
O	-2.67964800	-1.11393400	-0.41063100
C	-3.68418600	-1.66161300	-1.25021800
H	-3.52681700	-2.72621200	-1.43302800
H	-4.62836200	-1.53039200	-0.72406100
H	-3.72389200	-1.12203700	-2.20346800
C	-0.96078900	-3.62859700	-0.83447200
O	-1.91980900	-4.19259300	-0.39964000
O	-0.22249700	-4.13574500	-1.82818600
H	0.54161500	-3.55961700	-1.97805700

Elimination (4-methoxy-5-carboxy-β-D-xylopyranose ⟶ AXPL4 + H_2O)

Product (AXPL4)

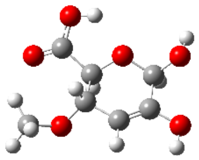

C	-1.28330100	-0.91762900	-0.56742800
C	-0.45096700	-2.00826500	0.13376800
C	1.51451400	-0.65653200	0.35446600

C	0.64307900	0.53867600	0.06154900
C	-0.60529500	0.41233400	-0.37808500
H	-0.64782200	-1.93956900	1.21003400
H	-1.37201100	-1.15437900	-1.63709400
H	1.62573100	-0.76588900	1.44426400
H	-1.22202600	1.28710400	-0.56178300
O	0.92489800	-1.83968800	-0.15688000
O	2.73819900	-0.48508700	-0.26095100
H	3.36603400	-1.11083000	0.11042600
O	1.27943500	1.69573000	0.35593200
H	0.67707500	2.43615100	0.23462300
O	-2.56263000	-0.84525900	0.01936500
C	-3.63101500	-1.30406700	-0.79264200
H	-3.52588500	-2.36494400	-1.02793900
H	-4.54112200	-1.15528500	-0.21420200
H	-3.69477600	-0.71823000	-1.71691700
C	-0.84793800	-3.40682000	-0.33557600
O	-1.91799000	-3.88057300	-0.09172300
O	0.06679700	-4.04509300	-1.06927400
H	0.86220100	-3.49338500	-1.12152900

TS-AXPL4

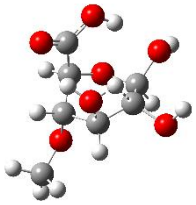

C	-1.67079200	-1.47418000	0.09004800
C	-0.60258100	-2.51808000	0.41644900
C	0.90001200	-0.81439500	1.22941700
C	0.36775700	0.02117100	0.09797800
C	-0.99853600	-0.17899900	-0.34404400
H	-1.10710300	-3.39563300	0.82781500
H	-2.31982400	-1.86995300	-0.69960200
H	0.82092000	-0.28061700	2.18144800
H	0.35418900	-0.34061600	-1.46731700
H	-1.64725400	0.69200100	-0.38062000
O	0.26361700	-2.06653300	1.42159000
O	2.27933700	-1.07105100	0.96858200
H	2.71592800	-1.26893900	1.80375300
O	0.69981700	1.38328700	0.30510700
H	1.53354500	1.55666900	-0.13537200
O	-0.65069600	-0.33866300	-1.97293100
H	-0.87448300	-1.20950800	-2.35281800
O	-2.40021300	-1.24844800	1.26801000
C	-3.68937300	-0.70957700	1.05043800
H	-4.29057200	-1.38008500	0.42607900
H	-4.15672900	-0.61204700	2.02821900
H	-3.64723100	0.27768800	0.57904700
C	0.10784300	-2.98659700	-0.86394600
O	-0.53726300	-3.27981600	-1.84103200

O	1.42419500	-3.06823300	-0.84629500
H	1.81233400	-2.60020800	-0.08026900

Elimination (4-methoxy-5-carboxy-β-D-xylopyranose ⟶ AXPL5 + H_2O)

Product (AXPL5)

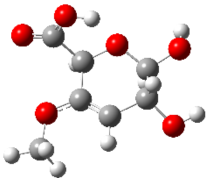

C	-1.30421800	-1.25740600	-0.21269500
C	-0.33025300	-2.29892300	0.31014800
C	1.29207600	-0.55739500	0.32716400
C	0.55349900	0.30948600	-0.67436800
C	-0.89567600	-0.07681500	-0.68360200
H	-0.51261300	-2.46255200	1.37808800
H	0.96001600	-0.31192400	1.34631000
H	1.01859200	0.14727600	-1.65677200
H	-1.58785000	0.66646000	-1.05658700
O	1.01203400	-1.92463300	0.06497100
O	2.65011200	-0.35082500	0.15877600
H	3.13516100	-0.83188500	0.83597400
O	0.64441000	1.67171200	-0.30919400
H	1.57224300	1.92477900	-0.35103900
O	-2.56851100	-1.69932900	-0.10859700
C	-3.59429800	-0.81796000	-0.52707000
H	-3.49010600	-0.58404900	-1.59042900
H	-4.53017600	-1.34261900	-0.35473200
H	-3.56918800	0.10675200	0.05684600
C	-0.56023500	-3.64543800	-0.39758000
O	-1.37806700	-4.43076900	-0.03418700
O	0.21729000	-3.84340800	-1.46727500
H	0.87959200	-3.13708700	-1.50158100

TS-AXPL5

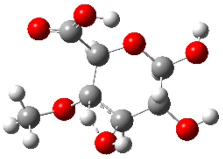

C	-1.30148400	-0.97350100	-0.19363000
C	-0.38799700	-2.10378400	0.29286300
C	1.35825000	-0.52054400	0.61108000
C	0.76426300	0.49270300	-0.35840700
C	-0.73635200	0.35555900	-0.28319400
H	-0.63399700	-2.32560600	1.33924500

H	-1.48755200	-0.42478100	-1.69949700
H	0.98243800	-0.36682500	1.63322400
H	1.13890100	0.19193700	-1.34653200
H	-1.22664700	1.15738800	0.26321000
O	1.00256300	-1.80729900	0.16237700
O	2.73595100	-0.38121800	0.54043000
H	3.15331500	-0.97419700	1.17264000
O	1.12275200	1.81489500	-0.05563300
H	2.08343600	1.84970800	0.01217700
O	-1.35381300	0.68701100	-1.83802100
H	-0.73752800	1.00767000	-2.51676600
O	-2.51633700	-1.00293900	0.51211100
C	-3.64492000	-1.41652900	-0.24378000
H	-3.49146700	-2.41375000	-0.66096200
H	-4.48906600	-1.43668900	0.44541900
H	-3.85498300	-0.70273000	-1.04795600
C	-0.65238800	-3.37090500	-0.51837000
O	-1.64526900	-4.02087600	-0.37807100
O	0.27762300	-3.67463600	-1.43298900
H	1.02222300	-3.06559100	-1.32249800

Elimination (4-methoxy-5-carboxy-β-D-xylopyranose ⟶ AXPL6 + CH_3OH)

Product (AXPL6)

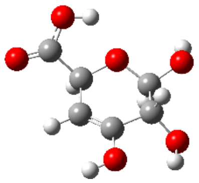

C	-0.96285200	-0.74708900	-0.18746600
C	-0.12623000	-1.94986500	0.15841200
C	1.79204500	-0.53730400	0.19436100
C	1.11325900	0.60621600	-0.54225300
C	-0.38103800	0.39748800	-0.52875000
H	-0.20175200	-2.18223300	1.22967200
H	-2.03946100	-0.87639000	-0.16363800
H	1.63350700	-0.41047000	1.27513800
H	1.47843900	0.61542000	-1.57781700
O	1.22890800	-1.77948800	-0.20890400
O	3.12720200	-0.54553000	-0.14347200
H	3.60012700	-1.14246100	0.44318900
O	1.46170000	1.79346300	0.12986100
H	0.90355900	2.50186400	-0.20615600
O	-1.02156900	1.54043300	-0.89392500
H	-1.97555000	1.41832200	-0.86630200
C	-0.67207400	-3.17245100	-0.58926300
O	-1.77554800	-3.58118000	-0.38121500
O	0.15300200	-3.71730600	-1.48003600
H	0.98262500	-3.21437700	-1.47844100

Product (CH_3OH)

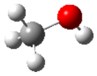

H	-2.28324100	-1.16175200	0.08977100
O	-3.18987700	-0.97679300	0.34082400
C	-4.04685100	-1.37879000	-0.71031200
H	-3.97335800	-2.45243800	-0.91203400
H	-5.06402700	-1.15806300	-0.39002800
H	-3.84844800	-0.82741500	-1.63522400

TS-AXPL6

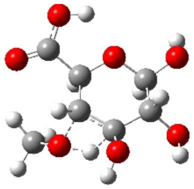

C	-1.34602300	-0.56981800	-0.13067600
C	-0.40662700	-1.64019400	0.41934100
C	1.48878100	-0.24897700	0.14240700
C	0.72836000	0.83098200	-0.60385800
C	-0.76157100	0.71114900	-0.40479600
H	-0.35941300	-1.56685200	1.51402000
H	-2.18623400	-0.91873000	-0.72400700
H	1.44066000	-0.06361200	1.22684000
H	0.96290700	0.70898900	-1.67179500
H	-1.46604400	0.82212200	1.00218500
O	0.88665300	-1.52187900	-0.12034300
O	2.78401000	-0.29372200	-0.32012300
H	3.32264600	-0.81168200	0.28388200
O	1.23360100	2.07208200	-0.14840200
H	0.73607800	2.75835400	-0.60200400
O	-1.41980600	1.49141000	-1.39253100
H	-2.10055500	2.02000600	-0.97353600
O	-2.23881600	0.00903400	1.23677200
C	-2.55777400	-0.66826700	2.45990800
H	-2.77899600	-1.70826200	2.21394300
H	-1.72265700	-0.61538900	3.16202400
H	-3.43769900	-0.20028000	2.90119700
C	-0.93944900	-3.04012600	0.11226100
O	-2.02472500	-3.38123700	0.48347000
O	-0.12161900	-3.82759700	-0.57594500
H	0.68589200	-3.33022100	-0.78357500

Elimination (4-methoxy-5-carboxy-β-D-xylopyranose ⟶ AXPL7 + CH_3OH)

Product (AXPL7)

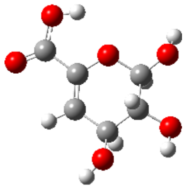

C	-0.83943400	-0.81708300	-0.35204400
C	-0.00993100	-1.84436800	-0.50729400
C	1.90964300	-0.54957200	0.09032500
C	1.15892800	0.64601700	-0.47126200
C	-0.31200200	0.56171900	-0.08395900
H	-1.90370300	-1.00503700	-0.42580900
H	1.80282900	-0.58910900	1.18265500
H	1.23504800	0.60830300	-1.56626500
H	-0.39414900	0.80054100	0.98552500
O	1.35031000	-1.76958300	-0.43671500
O	3.22200500	-0.48950200	-0.30487300
H	3.75490800	-1.07277600	0.24173400
O	1.74819400	1.80678200	0.05155900
H	1.23857700	2.55550900	-0.27529300
O	-0.95970500	1.56915200	-0.84521100
H	-1.86608000	1.67066900	-0.54362200
C	-0.50267700	-3.23488600	-0.80844700
O	-1.66322600	-3.50929900	-0.87347000
O	0.46383800	-4.13842300	-1.00229800
H	1.32570100	-3.70757900	-0.91592400

TS-AXPL7

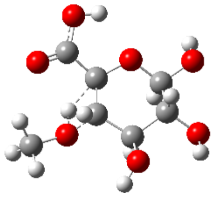

C	-0.91930900	-0.95577100	-0.56657500
C	-0.02727400	-2.12053100	-0.29519500
C	1.90894400	-0.74724200	-0.03315600
C	1.21297300	0.40971900	-0.73941800
C	-0.26363200	0.40241700	-0.35732000
H	-1.18551400	-1.94381600	0.93312100
H	-1.56480300	-0.99619300	-1.44884300
H	1.74623100	-0.68338200	1.05542200
H	1.33012200	0.25461100	-1.82010000
H	-0.30266800	0.63061400	0.71420600
O	1.36262000	-1.95442800	-0.51317700
O	3.24921700	-0.71703000	-0.36021600
H	3.70430600	-1.39784900	0.14313600
O	1.79207700	1.61857200	-0.32257900
H	1.30414800	2.32999700	-0.74961300
O	-0.88941100	1.43620400	-1.09822300
H	-1.65747800	1.75917400	-0.62121000
O	-1.88254600	-1.16983700	0.62210900
C	-3.27513400	-1.50351700	0.36567900
H	-3.31766800	-2.28960100	-0.38517900
H	-3.68354700	-1.83665600	1.31669000
H	-3.77336900	-0.59132000	0.04424000
C	-0.50386500	-3.37670900	-0.86124300
O	-1.67936500	-3.61148400	-1.07107700
O	0.43123500	-4.31256800	-1.09861800
H	1.29997300	-3.90982400	-0.96208200

Table S1. Heat capacity, *Cv*, in kcal mol^{-1} K^{-1} at different temperatures of chemical species involved in the initial elementary reactions: ring-opening, ring-contraction, and elimination.

Species	T/K 200	300	400	500	600	700	800	900	1 000
Xylose	0.02737	0.03831	0.04876	0.05790	0.06541	0.07151	0.07652	0.08071	0.08427
DXP	0.03046	0.04078	0.05065	0.05928	0.06640	0.07222	0.07705	0.08111	0.08458
FF1	0.02369	0.03236	0.04109	0.04882	0.05523	0.06046	0.06478	0.06840	0.07146
FF2	0.02393	0.03248	0.04113	0.04883	0.05523	0.06047	0.06479	0.06841	0.07147
AXP1	0.02345	0.03295	0.04186	0.04954	0.05581	0.06089	0.06506	0.06854	0.07149
AXP2	0.02340	0.03293	0.04191	0.04961	0.05588	0.06095	0.06511	0.06858	0.07152
AXP3	0.02282	0.03247	0.04153	0.04931	0.05565	0.06077	0.06498	0.06849	0.07146
AXP4	0.02507	0.03464	0.04364	0.05137	0.05768	0.06280	0.06699	0.07050	0.07346
AXP5	0.02297	0.03265	0.04166	0.04940	0.05571	0.06083	0.06502	0.06852	0.07149
AXP6	0.02332	0.03282	0.04177	0.04948	0.05577	0.06087	0.06506	0.06856	0.07152
AXP7	0.02327	0.03281	0.04186	0.04963	0.05593	0.06102	0.06518	0.06865	0.07158
H_2O	0.00596	0.00602	0.00617	0.00640	0.00665	0.00691	0.00717	0.00744	0.00771
2-O-acetyl-xylose	0.03607	0.04937	0.06243	0.07389	0.08335	0.09107	0.09743	0.10276	0.10728
OADXP	0.03936	0.05217	0.06451	0.07536	0.08440	0.09184	0.09803	0.10324	0.10768
FFL1	0.02357	0.03230	0.04105	0.04879	0.05520	0.06045	0.06477	0.06839	0.07145
FFL2	0.02379	0.03243	0.04116	0.04889	0.05528	0.06051	0.06483	0.06843	0.07149
AXPL1	0.03217	0.04405	0.05550	0.06547	0.07370	0.08043	0.08598	0.09062	0.09455
AXPL2	0.02338	0.03292	0.04190	0.04961	0.05588	0.06095	0.06511	0.06858	0.07152
AXPL3	0.02270	0.03243	0.04160	0.04941	0.05574	0.06084	0.06502	0.06851	0.07147
AXPL4	0.03437	0.04607	0.05747	0.06745	0.07568	0.08242	0.08797	0.09262	0.09656
AXPL5	0.03189	0.04380	0.05533	0.06537	0.07363	0.08037	0.08592	0.09057	0.09451
AXPL6	0.03146	0.04350	0.05514	0.06524	0.07354	0.08030	0.08587	0.09053	0.09448
AXPL7	0.03058	0.04292	0.05485	0.06512	0.07350	0.08029	0.08587	0.09052	0.09446
AA	0.01069	0.01426	0.01778	0.02087	0.02347	0.02564	0.02748	0.02905	0.03040
4OMGLA	0.03863	0.05270	0.06623	0.07809	0.08787	0.09584	0.10240	0.10788	0.11251
DGLA	0.04008	0.05425	0.06754	0.07908	0.08860	0.09638	0.10281	0.10821	0.11278
FFL1	0.03425	0.04640	0.05834	0.06886	0.07758	0.08472	0.09060	0.09552	0.09966
FFL2	0.03450	0.04650	0.05838	0.06888	0.07759	0.08473	0.09062	0.09553	0.09968
AXPL1	0.03431	0.04717	0.05925	0.06969	0.07825	0.08521	0.09093	0.09570	0.09972
AXPL2	0.03447	0.04732	0.05942	0.06986	0.07841	0.08534	0.09104	0.09579	0.09981
AXPL3	0.03357	0.04656	0.05882	0.06938	0.07802	0.08504	0.09081	0.09561	0.09966
AXPL4	0.03662	0.04928	0.06130	0.07172	0.08028	0.08724	0.09297	0.09774	0.10177
AXPL5	0.03381	0.04683	0.05903	0.06954	0.07815	0.08514	0.09088	0.09566	0.09970
AXPL6	0.03035	0.04241	0.05326	0.06233	0.06960	0.07542	0.08013	0.08403	0.08730
AXPL7	0.03109	0.04292	0.05360	0.06258	0.06978	0.07555	0.08023	0.08410	0.08735
Methanol	0.00747	0.00880	0.01071	0.01271	0.01455	0.01616	0.01757	0.01882	0.01992

Table S2. Entropy, *S*, in kcal mol^{-1} K^{-1} at different temperatures of chemical species involved in the initial elementary reactions: ring-opening, ring-contraction, and elimination.

Species	T/K 200	300	400	500	600	700	800	900	1 000
Xylose	0.08125	0.09491	0.10771	0.11985	0.13130	0.14202	0.15205	0.16144	0.17025
DXP	0.08790	0.10207	0.11505	0.12720	0.13856	0.14917	0.15907	0.16833	0.17700
FF1	0.08027	0.09160	0.10218	0.11224	0.12176	0.13070	0.13909	0.14695	0.15434
FF2	0.08114	0.09246	0.10300	0.11302	0.12250	0.13142	0.13978	0.14762	0.15498
AXP1	0.07714	0.08904	0.10015	0.11065	0.12050	0.12970	0.13829	0.14632	0.15384
AXP2	0.07756	0.08944	0.10057	0.11108	0.12095	0.13017	0.13877	0.14680	0.15432
AXP3	0.07713	0.08869	0.09963	0.11001	0.11978	0.12893	0.13748	0.14547	0.15296
AXP4	0.02507	0.03464	0.04364	0.05137	0.05768	0.06280	0.06699	0.07050	0.07346
AXP5	0.07682	0.08857	0.09962	0.11008	0.11992	0.12912	0.13770	0.14573	0.15325
AXP6	0.07719	0.08897	0.10001	0.11045	0.12027	0.12944	0.13801	0.14602	0.15353
AXP7	0.07780	0.08950	0.10052	0.11096	0.12078	0.12996	0.13852	0.14653	0.15403
H_2O	0.04189	0.04512 (0.04516)	0.04744 (0.04749)	0.04929 (0.04935)	0.05084 (0.05090)	0.05219 (0.05226)	0.05339 (0.05348)	0.05449 (0.05458)	0.05549 (0.05561)
2-O-acetyl-xylose	0.09548	0.11204	0.12762	0.14247	0.15652	0.16972	0.18210	0.19371	0.20460
OADXP	0.09922	0.11685	0.13300	0.14814	0.16232	0.17559	0.18799	0.19959	0.21048
FFL1	0.08007	0.09136	0.10193	0.11199	0.12150	0.13045	0.13883	0.14670	0.15409
FFL2	0.08090	0.09224	0.10282	0.11289	0.12241	0.13135	0.13974	0.14760	0.15498
AXPL1	0.09098	0.10579	0.11969	0.13288	0.14533	0.15701	0.16794	0.17818	0.18780
AXPL2	0.07758	0.08946	0.10058	0.11109	0.12096	0.13017	0.13877	0.14681	0.15433
AXPL3	0.07809	0.08945	0.10027	0.11058	0.12030	0.12939	0.13789	0.14584	0.15329
AXPL4	0.03437	0.04607	0.05747	0.06745	0.07568	0.08242	0.08797	0.09262	0.09656
AXPL5	0.09121	0.10588	0.11970	0.13285	0.14527	0.15692	0.16784	0.17807	0.18767
AXPL6	0.09053	0.10508	0.11885	0.13199	0.14440	0.15606	0.16698	0.17722	0.18683
AXPL7	0.08916	0.10359	0.11736	0.13055	0.14303	0.15475	0.16573	0.17602	0.18567
AA	0.06251	0.06805 (0.06762)	0.07302 (0.07272)	0.07762 (0.07741)	0.08190 (0.08176)	0.08589 (0.08581)	0.08961 (0.08958)	0.09309 (0.09309)	0.09636 (0.09639)
4OMGLA	0.09739	0.11519	0.13182	0.14759	0.16245	0.17638	0.18942	0.20163	0.21308
DGLA	0.09994	0.11812	0.13501	0.15090	0.16581	0.17975	0.19277	0.20495	0.21638
FFL1	0.09527	0.11065	0.12507	0.13880	0.15177	0.16396	0.17539	0.18610	0.19617
FFL2	0.09565	0.11107	0.12548	0.13918	0.15214	0.16431	0.17573	0.18643	0.19649
AXPL1	0.09265	0.10863	0.12358	0.13772	0.15101	0.16344	0.17505	0.18591	0.19609
AXPL2	0.09305	0.10917	0.12422	0.13844	0.15180	0.16428	0.17594	0.18683	0.19704
AXPL3	0.09289	0.10853	0.12331	0.13734	0.15056	0.16295	0.17453	0.18536	0.19552
AXPL4	0.03662	0.04928	0.06130	0.07172	0.08028	0.08724	0.09297	0.09774	0.10177
AXPL5	0.09217	0.10806	0.12299	0.13713	0.15044	0.16289	0.17452	0.18540	0.19560
AXPL6	0.08763	0.10211	0.11572	0.12852	0.14048	0.15159	0.16192	0.17153	0.18051
AXPL7	0.08862	0.10337	0.11710	0.12997	0.14196	0.15310	0.16344	0.17307	0.18205
Methanol	0.05294	0.05700 (0.05736)	0.06036 (0.06062)	0.06340 (0.06358)	0.06625 (0.06633)	0.06892 (0.06892)	0.07143 (0.07137)	0.07381 (0.07369)	0.07606 (0.07588)

The experiment-derived values of water, acetic acid, and methanol are provided in round brackets. See Stull et al.[42]

Table S3. $\mathcal{T}_1$ diagnostic.

Species	$\mathcal{T}_1$
Xylose	0.011
DXP	0.012
TS-DXP	0.012
FF1	0.012
TS-FF1	0.016
FF2	0.013
TS-FF2	0.016
AXP1	0.012
TS-AXP1	0.016
AXP2	0.012
TS-AXP2	0.015
2-O-acetyl-xylose	0.013
OADXP	0.014
TS-OADXP	0.014
AXPL1	0.013
TS-AXPL1	0.015
AXPL2	0.014
TS-AXPL2	0.015
4-O-methyl-D-glucuronic acid	0.013
DGLA	0.014
TS-DGLA	0.015
FFL1	0.014
TS-FFL1	0.014
AXPL1	0.014
TS-AXPL1	0.014
AXPL2	0.013
TS-AXPL2	0.015
